# High-resolution precipitation monitoring with a dense seismic nodal array

**DOI:** 10.1038/s41598-023-38008-w

**Published:** 2023-07-15

**Authors:** Junlin Hua, Mengxi Wu, Jake P. Mulholland, J. David Neelin, Victor C. Tsai, Daniel T. Trugman

**Affiliations:** 1 Department of Geological Sciences, Jackson School of Geosciences, The University of Texas at Austin, Austin, TX 78712 USA; 2 Joint Institute for Regional Earth System Science and Engineering, University of California, Los Angeles, Los Angeles, CA 90095 USA; 3 Department of Atmospheric Sciences, John D. Odegard School of Aerospace Sciences, The University of North Dakota, Grand Forks, ND 58202 USA; 4 Department of Atmospheric and Oceanic Sciences, University of California, Los Angeles, Los Angeles, CA 90095 USA; 5 Department of Earth, Environmental and Planetary Sciences, Brown University, Providence, RI 02912 USA; 6 Nevada Seismological Laboratory, University of Nevada, Reno, Reno, NV 89557 USA

**Keywords:** Seismology, Natural hazards, Atmospheric science

## Abstract

Accurate precipitation monitoring is crucial for understanding climate change and rainfall-driven hazards at a local scale. However, the current suite of monitoring approaches, including weather radar and rain gauges, have different insufficiencies such as low spatial and temporal resolution and difficulty in accurately detecting potentially destructive precipitation events such as hailstorms. In this study, we develop an array-based method to monitor rainfall with seismic nodal stations, offering both high spatial and temporal resolution. We analyze seismic records from 1825 densely spaced, high-frequency seismometers in Oklahoma, and identify signals from nine precipitation events that occurred during the one-month station deployment in 2016. After removing anthropogenic noise and Earth structure response, the obtained precipitation spatial pattern mimics the one from a nearby operational weather radar, while offering higher spatial (~ 300 m) and temporal (< 10 s) resolution. We further show the potential of this approach to monitor hail with joint analysis of seismic intensity and independent precipitation rate measurements, and advocate for coordinated seismological-meteorological field campaign design.

## Introduction

Accurate monitoring of precipitation is essential to our understanding of the water and energy cycles, and can inform rainfall-driven hazard mitigation. Surface precipitation can be used to infer information about atmospheric water vapor, convection and latent heating, and it is a key component of the water budget for terrestrial ecological and hydrological modeling^1, 2^. Regarding hazards, extreme precipitation can cause mass movements including landslides and debris flows^3^, and produce flash floods when the precipitation rate exceeds the infiltration capacity^4^. Furthermore, long-term observational precipitation data facilitate studies of climate change^5^, which can have highly variable impacts at local scales.

Among these precipitation-related hazards, hailfall is known to cause severe economic damage and bodily injury. Hail often brings significant losses in both urban areas and farmland^6, 7^. One recorded hailstorm in 1995 injured 109 people during an outdoor festival^8^, and hailstorms may even cause deaths^9^. Therefore, accurate real-time quantification of the areal extent and intensity of hailfall is highly relevant for hazard mitigation.

Currently, precipitation is usually monitored in two ways: (1) direct measurement on the ground, or (2) remote sensing of hydrometeors (i.e., liquid and solid water particles in the air). Automatic direct measurement of surface rainfall is most commonly conducted using catching-type rain gauges, such as tipping-bucket gauges, which are globally used in weather stations^10^ to measure precipitation. Instrument sensitivity depends on bucket size (typically ~ 0.2 mm), and hence, the time interval for it to record new precipitation (i.e., the integration time between bucket tips) depends on the precipitation intensity^11^. Therefore, when precipitation rate is low, timely precipitation updates are not available, and when precipitation is high, gauges underestimate precipitation during emptying periods^11^. Furthermore, low-cost tipping-bucket gauges are not designed to measure droplet sizes. Unlike rain or snow, direct hail measurement still requires much human effort using disposable foam hailpads^12^, especially given that hailpad networks need to be dense because of the local character of hailfall^13–15^.

Unlike surface measurements which can only sample precipitation from a small areal extent, ground-based and space-borne radar is used to detect precipitation over large areas. Radar gains information about hydrometeors in the atmosphere and then estimates precipitation based on empirical relationships between reflectivity and precipitation rate^16, 17^, forward modeling of attenuation by hydrometeors^18^, or the shapes of raindrops measured by orthogonally polarized signals^19^. Weather radar can also provide probabilistic hail indices, such as probability of hail and maximum expected hail size^20^. However, while offering good spatial coverage, depending on the instruments and platforms, the temporal resolution of typical radar precipitation products is longer, ranging from minutes to hours, and satellite radar precipitation products have a lower spatial resolution (kilometer-scale). Satellite-based radar products may also come with latencies that limit their use in real-time applications.

Recent advancements in understanding seismic precipitation signals^21–23^ provide an alternative to counter these weaknesses of existing precipitation monitoring approaches by measuring seismic waves. Seismic precipitation signals are generated when raindrops impact the ground and excite waves, at frequencies typically above 50 Hz^21–23^ for nearby raindrop impacts. Hence, unlike remotely-sensed radar measurements, the seismic intensity serves as a direct sampling of surface precipitation similar to rain gauges with high temporal resolution. Meanwhile, compared to traditional tipping-bucket rain gauges, the seismic intensity is dependent on the weight and speed of each raindrop in addition to the overall precipitation rate^21–23^, making it sensitive to precipitation type and hydrometeor (e.g., droplet) size, and thus could potentially be used to detect hail. Because a single seismic station is sensitive only to raindrops that fall within ~ 10 m of it^21^, a seismic array is required to monitor regional rainfall patterns.

Oklahoma is a well-suited place to test the proposed seismic array precipitation monitoring approach. The climate in Oklahoma is regulated by low-level warm and moist advection from the Gulf of Mexico and mid-level cold and dry air from Canada and the Rocky Mountains, which bring severe weather to the southern Great Plains. Thunderstorms frequently occur between April and October, peaking in May and June, and are often accompanied by tornadoes and large hail^24^. A low-level jet stream flows from the Gulf of Mexico through parts of Oklahoma, overlapping with locations that experience the most severe weather^25^. Central and North Central Oklahoma display two precipitation peaks throughout the year, in May and September^24^.

**Figure 1:**
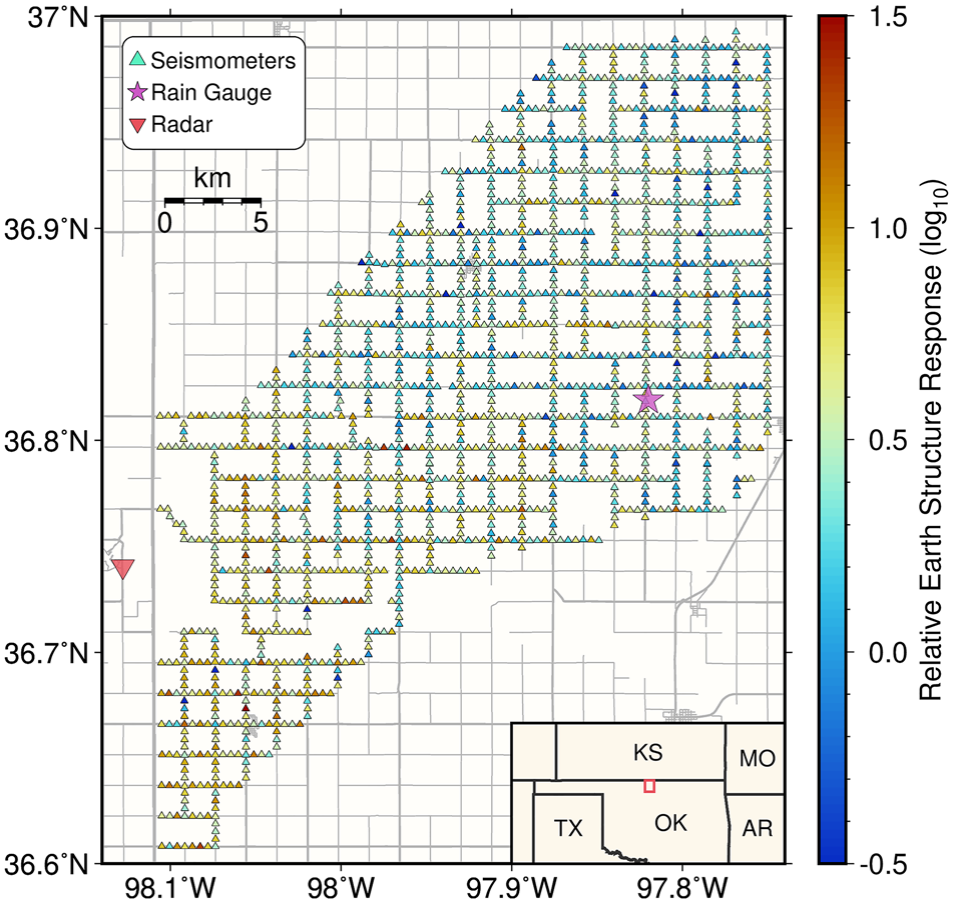
Map of the study area. Triangles show individual seismic nodal stations, color coded by the relative Earth structure response with respect to the reference station (Network: 2A, Station: 340, location shown in Fig. S1). The inverted red triangle and the magenta star mark locations for the ground-based radar and the rain gauge, respectively. Gray lines are roads. The study area is outlined by the red box in the map at the bottom right corner. The software used to create this map is the Generic Mapping Tools, version 6.4.0^49^ (https://www.generic-mapping-tools.org).

Between 14 April 2016 and 10 May 2016, 1833 high-frequency seismic nodal stations from the LArge-n Seismic Survey in Oklahoma (LASSO) experiment were deployed with nominal station spacing of ~ 400 m along county roads in Grant County, Oklahoma (Fig. 1), for a study region approximately 25 km by 32 km. These nodal stations have a sampling rate of 500 Hz, and were buried in ~ 18 cm-deep holes with ~ 3 cm soil cover^26^. Such shallow burial depths enable detection of rainfall signals^23^. Compared to broadband seismometers, these seismic nodes can only record high frequency vertical vibrations but are much cheaper, with a cost comparable to a tipping-bucket rain gauge. Currently designed mainly for temporary experiments, their power is supplied by batteries with data being stored locally. However, since seismic data are digitized, nodes can be designed to transmit data in real-time at similar or lower cost if both electricity and data transmission are accessible. Though the experiment was initially designed to study the induced seismicity around the region^27^, this array offers a unique opportunity for retrieving seismic rainfall signals with spatial structures.

During the deployment period, there were nine precipitation events in the same region, with each event consisting of multiple sub-events separated by short breaks. These events pertain to different storm types (Table S1), including disorganized “pulse-type” thunderstorms, supercell thunderstorms, and mesoscale convective systems with scattered instances of large hail, and thus form an ideal dataset to test the seismic precipitation monitoring approach.

In this study, we extracted seismic precipitation signals from all LASSO stations. We then solved the two main challenges for array-based monitoring: (1) removing anthropogenic noise, and (2) accounting for differences in Earth structure response between stations. With these corrections, seismic-estimated precipitation intensities from the array are compared with measurements from a local tipping-bucket rain gauge at a Department of Energy (DOE) Atmospheric Radiation Measurement (ARM) external facility and a nearby WSR-88D S-band ground-based operational weather radar at Vance Air Force Base, Oklahoma (Fig. 1), for validation. In addition to seismic-only precipitation rate retrievals, we also performed a joint analysis of seismic intensities and radar precipitation rate products to test the potential use for hail detection.

## Results

### Seismic-derived precipitation signal and its physical meaning

Based on satellite and radar data for the study area, nine precipitation events occurred during the deployment period and within the footprint of the array (with each event containing multiple sub-events). Seismograms were requested for all these events from the IRIS DMC, and we obtained seismic waveform data from 1825 individual stations (Fig. 1) (data were not sufficiently recovered for eight of the deployed stations).

**Figure 2:**
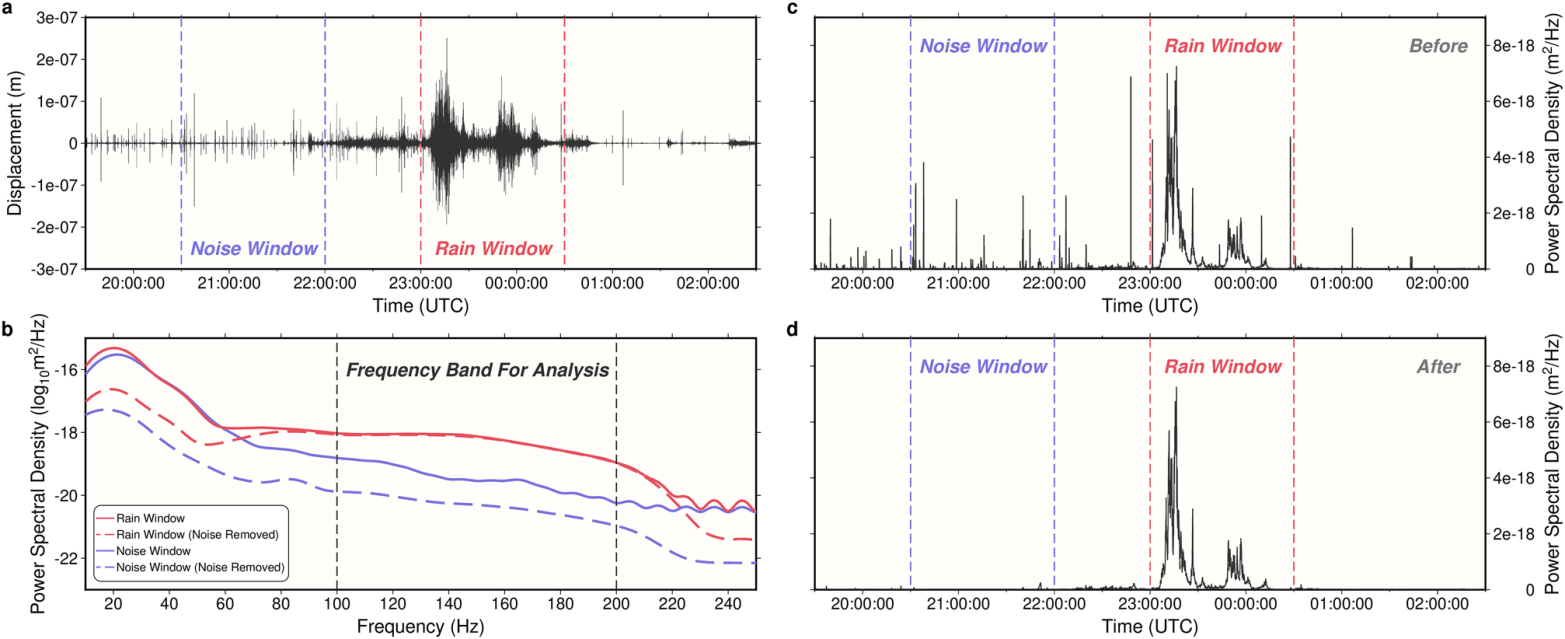
Seismic precipitation signals. (**a**) Displacement seismogram for a precipitation event on 19 April 2016 sampled by Station: 150 (black line), filtered at 100–200 Hz. Rain window (red) and noise window (blue) for following analyses are divided by dashed lines. (**b**) Averaged power spectral density for the rain window (red) and noise window (blue) in (**a**). Solid lines show original PSD, dashed lines show denoised PSD. The frequency range between 100 and 200 Hz is less affected by noise. (**c**) and (**d**) Averaged seismic power spectral density between 100 and 200 Hz before and after removing anthropogenic noise, respectively. Times used in this study are all in UTC.

Based on the seismograms, a much higher level of background ‘tremor’ is observed during precipitation events (e.g., Fig. 2a), and these elevated tremor records are hereinafter referred to as seismic precipitation signals. Power spectral densities (PSDs) were calculated every second between 10 Hz (the corner frequency of the nodal instruments) and 250 Hz (Nyquist frequency) using Welch’s method^28^ (Methods). Welch’s method calculates the overall PSD around ± 5 s, making the effective time resolution of the PSD 1–10 s. Comparing seismic PSDs for time windows with and without precipitation, seismic power at frequencies over 60 Hz is greater during precipitation (Fig. 2b). This finding aligns with previous studies indicating that elevated seismic PSD appears above 50–80 Hz^21–23^.

These elevated seismic PSDs during precipitation are caused by raindrops (or hailstones) hitting the ground^21, 22^. As the seismic precipitation signal is due to the combination of seismic waves from all impact events between raindrops and the ground, the impact induced seismic ground motion can be modelled similarly to stochastic bedload impacts^21, 29^, and the recorded displacement spectrum *u*_*i*_(*f*) at station *i* at a distance of *r* can be characterized as:

1 $$u_{i} (f) = F_{j} (f)G_{i} (f,r),\quad F_{j} (f) = m_{j} v_{j} e^{{ - i2\pi ft_{j} }} ,$$

where *F*_*j*_(*f*) is the force of the impact (Fourier transform of $m_{j} v_{j} \delta (t - t_{j} )$, $\delta$ is the Dirac delta function), and *m*_*j*_, *v*_*j*_, and *t*_*j*_ are the mass, fall speed, and impact time of a single raindrop *j*. *G*_*i*_ (*f*, *r*) is the displacement Green’s function which represents the response of Earth structure to seismic wave propagation (meanings for all used symbols summarized in Table S2). This expression is valid when the impact is instantaneous, and the raindrop does not rebound. Assuming impacts happen randomly in space, the PSD of the displacement seismogram *PSD*_*i*_ (*f*) is expressed as:

2 $$PSD_{i} (f) = \left| {NF^{2} (f)} \right|\int\limits_{0}^{\infty } {2\pi r} G_{i}^{2} (f,r)dr = Nm^{2} v^{2} \int_{0}^{\infty } {2\pi rG_{i}^{2} (f,r)dr} = 2\rho_{w} \cdot PR_{i} \cdot E_{i} \cdot S_{i} ,$$

where *N* is the number of impacts per area per time^29^, *m* is the raindrop mass, and *v* is the raindrop terminal fall speed. *Nm* is equivalent to the raindrop density (*ρ*_*w*_) multiplied by the precipitation rate *PR*, 0.5 *mv*^2^ represents the kinetic energy of a raindrop particle (*E*), and *S* characterizes the remaining Earth structure response. Here, Eq. (2) is a simplified approximation valid when all raindrops have the same mass and fall speed. The full derivation for how seismic PSD is related to precipitation rate and kinetic energy with the consideration of raindrop size distribution is available in Methods, and the relationship is similar to Eq. (2). Therefore, an elevated PSD could indicate increases in the precipitation rate, the size of raindrops, or both, as the kinetic energy of raindrops increases with their size. This formulation applies to both raindrops and hailstones and thus lays the foundation to monitor both regular precipitation events (rain) and hailfall (often with higher fall speeds and thus kinetic energy) using seismic data analysis.

### Removing anthropogenic noise and Earth structure response

Based on this quantitative framework, we use the average seismic PSD between 100 and 200 Hz (Fig. 2b) to characterize precipitation strength (Fig. 2c). This frequency band is selected as it is above 60 Hz, where nearby precipitation starts to dominate the observed tremor, and below 220 Hz, where signals become very weak (Fig. 2b). The band is also higher than the main anthropogenic noise window^23^ (4–80 Hz), river noise window^21^ (< 100 Hz), and where wind noise potentially dominates^23^ (< 70 Hz). In addition, this band is above the corner frequency of all influential earthquakes^30, 31^. As expected, consistently high PSD amplitudes are observed during precipitation, but occasional high PSD pulses also occur in intervals without rain (Fig. 2c).

Non-precipitation PSD pulses were removed based on their common features. Since the nodal stations were often deployed along roads, the majority of these pulses are short duration traffic signals and are easily removed (Fig. 2c). Compared with precipitation signals, these anthropogenic pulses are also particularly strong at low frequencies^23^ (< 80 Hz, Fig. 2b). Based on these two characteristics, denoising criteria were designed to find and remove this anthropogenic noise (Methods). After denoising, most pulses were successfully removed (Fig. 2d), and as expected, seismic PSDs during non-precipitation intervals are significantly reduced and now two orders of magnitude lower than during precipitation intervals (Fig. 2b). We found denoising only marginally reduces the 100–200 Hz PSD during precipitation (Fig. 2b).

In order to analyze signals from different stations systematically, their different Earth structure responses^22, 23^ must be corrected. If detailed 3-D spatial maps of elastic moduli and density for the top 30 m around the region were available, the absolute Earth structure response could be estimated by numerically solving the seismic wavefield excited by raindrops impacting at any possible location. However, such detailed seismic structure is not available, and there are no methods available to infer this structure with sufficient detail. Therefore, here we estimate the relative Earth structure response between stations based directly on seismic precipitation signals, which does not require any prior knowledge. Based on Eq. (2), the difference in log-scale for seismic PSD from two stations *i* and *k* is expressed as:

3 $$\log \frac{{PSD_{i} }}{{PSD_{k} }} = \log \frac{{PR_{i} \cdot E_{i} \cdot S_{i} }}{{PR_{k} \cdot E_{k} \cdot S_{k} }} = \left( {\log PR_{i} \cdot E_{i} - \log PR_{k} \cdot E_{k} } \right) + \left( {\log S_{i} - \log S_{k} } \right).$$

The first part on the right-hand side of Eq. (3) corresponds to differences in rain intensity between stations, and the second part corresponds to their difference in Earth structure response. To quantify the Earth structure response, we first measured this seismic PSD difference ($\log \left( {{{PSD_{i} } \mathord{\left/ {\vphantom {{PSD_{i} } {PSD_{k} }}} \right. \kern-0pt} {PSD_{k} }}} \right)$) at station pairs that are within 1.5 km of each other, for each shared precipitation window when the PSD variations with time are similar between the stations (Methods), and calculated their average. Because only close station pairs were used, their overall precipitation intensity should be similar. Therefore, after averaging, the first part on the right-hand side of Eq. (3) is eliminated, with only the Earth structure difference remaining (second part of the right-hand side in Eq. 3). With this, we set the response at a reference station to be one (Station 340), and solved for the optimal relative Earth structure response *R* at each station to minimize an *L*_2_-norm cost function that is similar to: $\left\| {\log \left( {{{PSD_{i} } \mathord{\left/ {\vphantom {{PSD_{i} } {PSD_{k} }}} \right. \kern-0pt} {PSD_{k} }}} \right) - \log \left( {{{R_{i} } \mathord{\left/ {\vphantom {{R_{i} } {R_{k} }}} \right. \kern-0pt} {R_{k} }}} \right)} \right\|_{2}$ (Methods, Eq. 17). This optimization problem has an explicit and constant Hessian, so those relative responses *R* can be directly obtained using Newton’s method^32^ (Fig. 1, Fig. S1a), with their standard deviations provided by the inverse Hessian^33^ (Fig. S1b). More details about the optimization are available in Methods.

The relative structure response at different stations shows two orders of magnitude differences (Fig. 1), indicating a substantial difference in burial depth or soil type^23^, and emphasizing the importance of this correction. However, the low standard deviation (Fig. S1b) for the solved responses ensures the accuracy after correction. Interestingly, we also found the resolved structure response broadly similar to the spatial pattern of high-frequency seismic ground motion due to teleseismic waves^34^, again indicating the influence of near-surface lithology on the amplitude of seismic records. In the following analyses, seismic PSDs are divided by their relative Earth structure response *R*.

### Monitoring precipitation with seismic data

**Figure 3:**
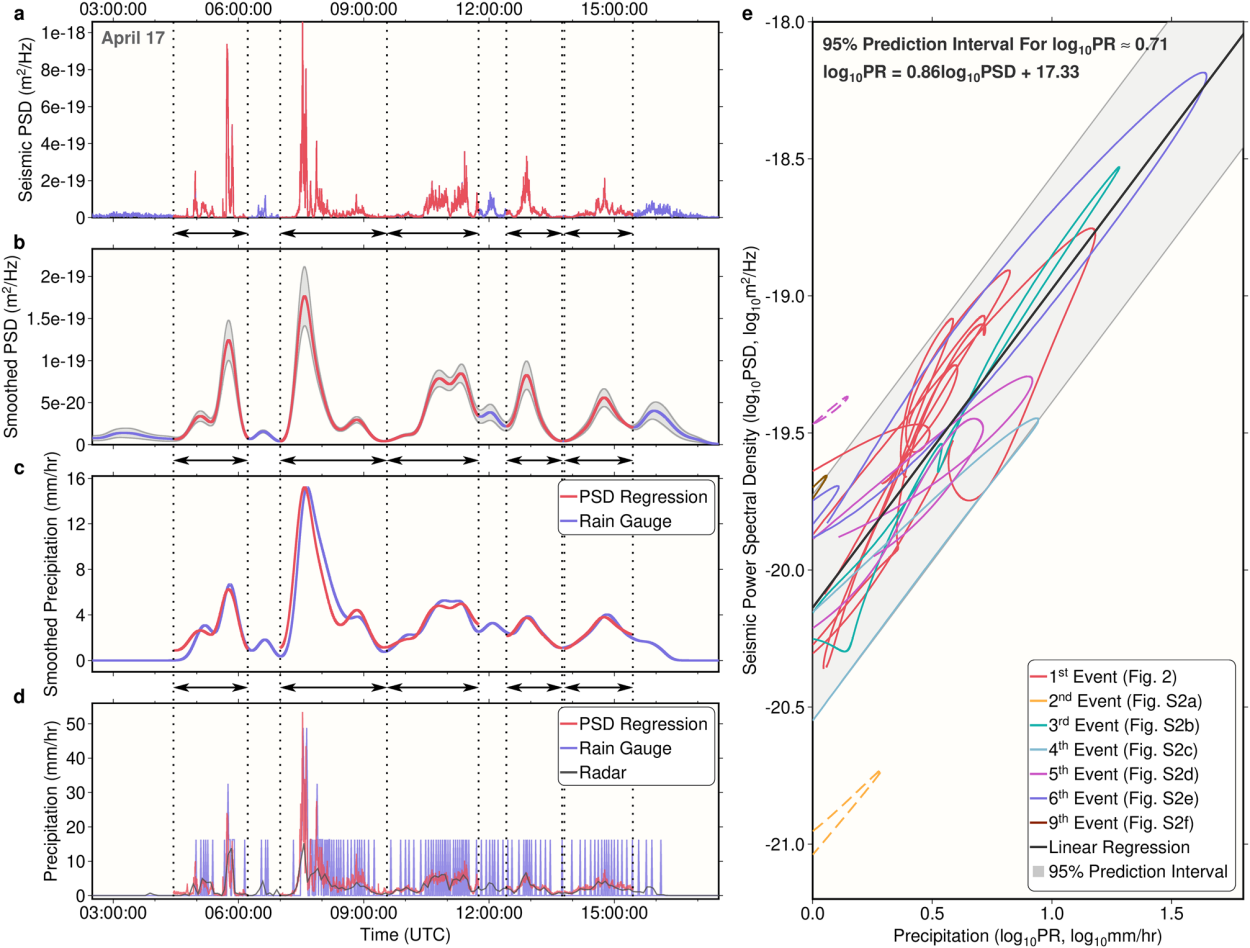
Seismic precipitation measurements compared to rain gauge data. (**a**) Seismic power spectral density at the location of the rain gauge for a precipitation event on 17 April 2016. The profile is obtained by the weighted average seismic PSD sampled at several nearby stations. Red sections mark five individual precipitation sub-events, and are divided by black dotted lines. (**b**) Similar to (**a**), but smoothed. The gray shadow shows one standard deviation due to station averaging. All smoothing for panels in this figure done by convolving a Gaussian (10 min half-width). (**c**) The blue line shows smoothed precipitation rate from the rain gauge. Red sections are converted precipitation rate from seismic PSD by fitting each red PSD section in (**b**)**.** to the rain gauge measurements in **c.** through linear regression after both are converted to log-scales. (**d**) The blue line shows raw rain gauge records, which often appear discretized due to the time intervals between bucket tips. Red lines show converted precipitation rate using the unsmoothed PSD in (**a**) and the regression relationship in (**c**). The gray line shows instantaneous precipitation rate from the ground-based radar. (**e**) Smoothed seismic PSD versus smoothed rain gauge precipitation rate in log-scale. Here, each line represents the evolution during an event. Different colors for events on different days (Fig. S1, each event may consist of multiple sub-events as separated lines). The black line shows the fitted linear relationship between the PSD and the precipitation rate using all sub-events except two dashed line outliers, and the 95% prediction interval for the fitting is characterized by the gray shadow. Fitted relationship and the 95% interval width are shown at the top left corner. Events 7 and 8 are not shown since they did not pass the rain gauge.

Seismic PSDs at each location are then calculated by the weighted average of seismic PSDs from stations that are less than 1.7 km away (Methods). To compare seismic-derived precipitation signals with other precipitation measurements, we first obtained the averaged PSD at the location of a tipping-bucket rain gauge (Fig. 1). The seismic-derived precipitation estimates clearly show elevated PSDs during precipitation periods (Fig. 3a, Fig. S2). An example is shown for the precipitation event on 17 April 2016, which consisted of five sub-events over ~ 12 h (Fig. 3a).

The seismic-derived signal for this event is compared with precipitation rate from the rain gauge, computed as the increased rainfall accumulation between time steps divided by the interval. Because the time between bucket tips for the rain gauge could be longer than one minute (the measurement interval), when the rain rate is low, the precipitation record is not always continuous (Fig. 3d, Fig. S2). Hence, for comparison, both seismic PSD and rain gauge precipitation rates were smoothed by convolving a 10-min half width Gaussian (Fig. 3b,c), and it is shown that both timing and relative strength were comparable between the two measurements for those precipitation sub-events.

To estimate the precipitation rate using seismic PSDs, we derived their conversion relationships. Based on Eq. (2), in log-scale, the seismic PSD (log*PSD*) varies linearly with the precipitation rate (log*PR*). For each sub-event, we independently obtained parameters to convert seismic PSD linearly to precipitation rate using the ordinary least-square method (Methods), and a close fit is reached (Fig. 3c, Fig. S2). These conversion parameters were then applied to the unsmoothed PSD as well (Fig. 3a). Compared with precipitation rates from the rain gauge and the nearby operational weather radar (Fig. 1), seismic PSD-derived precipitation rates offer better temporal resolution (Fig. 3d, Fig. S2).

The overall conversion relationship between seismic PSD and precipitation rate is also calculated in the same manner using data from all events (Methods). Based on this relationship (Fig. 3e), the *PSD* is linearly related to *PR*^1.16^, indicating a dependence of raindrop kinetic energy on precipitation rate (Eq. 2). This dependence is weaker than that from a previous study (*PR*^1.9^) in Southeastern France^21^ where rainfall receives Mediterranean influence^35^, potentially due to differences in the type of precipitating weather systems. The prediction interval of the relationship is relatively wide (Fig. 3e), suggesting raindrop kinetic energy varies between events. In particular, the first sub-event on 26 April 2016 (Fig. S2d) shows abnormally higher seismic PSD relative to the contemporary precipitation (Fig. 3e), indicating much larger kinetic energy for the falling raindrops or ice particles (i.e., hail); as will be discussed in following sections, this was likely a hailfall event. Another abnormal event is on 18 April 2016 where the PSD is very low.

Besides variations in raindrop kinetic energy, the relatively large prediction interval in Fig. 3e could also be caused by the time-lag between seismic PSDs and rain gauge precipitation (lines in Fig. 3e are often elliptical rather than straight). The time-lag is partly because no seismic station co-locates with the rain gauge, and the spatially averaged seismic PSD is only an approximation for the rain gauge location. It is also partly because the tipping time for the rain gauge lags the time when the rainwater was first collected, which makes the seismic PSD often lead the rain gauge precipitation (Fig. 3, Fig. S2), and this shows an advantage of the seismic PSD with higher temporal resolution.

**Figure 4:**
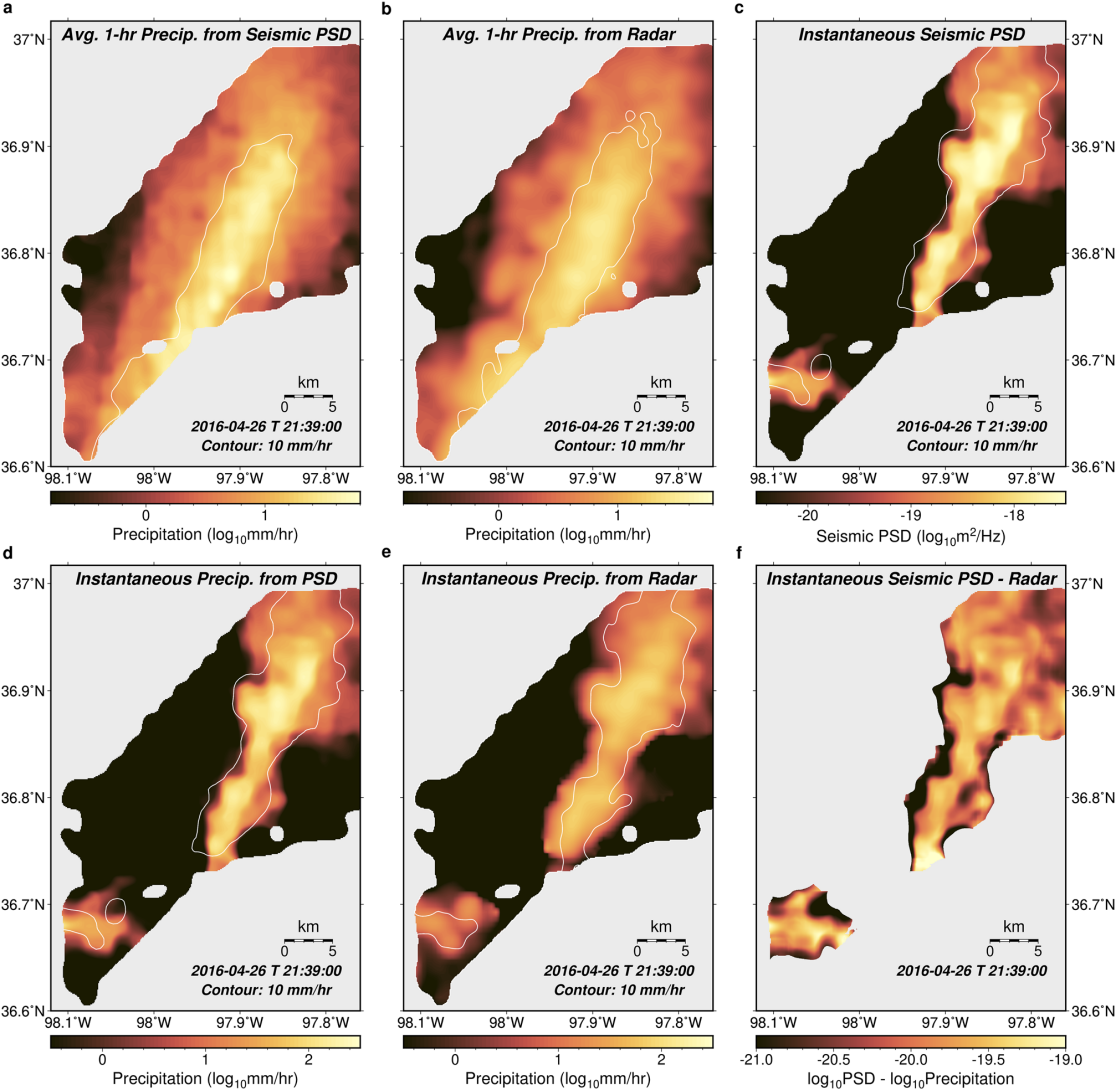
Precipitation spatial distribution. (**a**) and (**b**) Maps in log-scale for the hourly precipitation accumulation based on seismic power spectral density and weather radar at 21:39 UTC on 26 April 2016. The seismic PSD derived precipitation rate is based on the relationship in Fig. 3e. The white line in (**a**) shows the contour for 10 mm hr^−1^ radar precipitation in (**b**), and the white line in (**b**) shows the contour for 10 mm hr^−1^ seismic PSD precipitation in (**a**). Regions with low data coverage are shown in gray. (**c**)–(**e**) Maps in log-scale for instantaneous precipitation rate at the same time as (**a**) and (**b**), (**c**) shows the raw seismic PSD at different locations, and (**d**) converts (**c**) to precipitation rate based on the relationship in Fig. 3e. (**e**) shows the radar instantaneous precipitation rate. White lines in (**c**) and (**d**) are the contour for 10 mm hr^-1^ radar precipitation in (**e**), while the while line in (**e**) is the contour for 10 mm hr^-1^ seismic PSD precipitation in (**d**). (**f**) The seismic PSD map in (**c**) minus the radar precipitation map in (**e**) (PSD-PR difference). Only regions with both radar precipitation rate and seismic converted precipitation rate (Fig. 3e) higher than 0.3 mm hr^−1^ are plotted. The variable plotted in (**f**) is expected to be proportional to the kinetic energy of a raindrop. The software used to create these maps is the Generic Mapping Tools, version 6.4.0^49^ (https://www.generic-mapping-tools.org).

We then generated seismic precipitation maps for the entire region using the same weighted averaging method based on nearby stations (Methods). These maps are compared with both hourly precipitation accumulation and instantaneous precipitation rate retrieved from a nearby operational ground-based weather radar, whose close distance (Fig. 1) ensures a lateral resolution as high as ~ 300 m over the area (angular resolution of 1°). In general, precipitation patterns are similar (e.g., Fig. 4, Fig. S3) between the two measurements, but seismic maps show much higher temporal resolution (< 10 s vs. > 4 min, Movies S1–S9). Seismic maps also show narrower precipitation regions than the radar (e.g., Fig. 4a vs. b), suggesting a higher effective spatial resolution.

To further test the robustness of the seismic method, we statistically compared the precipitation map based on seismic PSD to that from the weather radar (Fig. S4). The result shows that for both hourly precipitation accumulation and instantaneous precipitation rate, the spatial correlation coefficients between seismic and radar maps are around 0.75–0.8, and the overall difference in estimated precipitation rate is around 1–2 mm h^−1^, with the seismic PSD slightly underestimating the precipitation rate compared to radar data. Given that the conversion relationship in Fig. 3e is based on rain gauge measurements for a limited number of events and the influence of raindrop kinetic energy is ignored, the seismic method performs reasonably well. We also generated heatmaps of intense rainfall with instantaneous precipitation rate over 25 mm h^−1^ (Fig. S4) based on seismic PSD and weather radar, and the result shows good agreement between the two approaches, with the seismic PSD indicating a narrower region with frequent intense rain rate during the study period, possibly reflecting a higher effective spatial resolution than the radar.

### Possible hail detection through joint analyses of seismic and precipitation measurements

Seismic signals are sensitive to the particle kinetic energy, so hailfall can potentially be monitored by combining seismic PSD with independent precipitation rate measurements. Based on Eq. (2), the difference between the PSD and the precipitation rate (hereinafter referred to as the PSD-PR difference), defined as log*PSD–*log*PR*, is proportional to the particle kinetic energy (log*E*). Therefore, the difference between a seismic PSD map and an independent precipitation rate map (here we use instantaneous precipitation from radar measurements) would indicate the kinetic energy of hydrometeors (Fig. 4c, Movies S1–S9).

**Figure 5:**
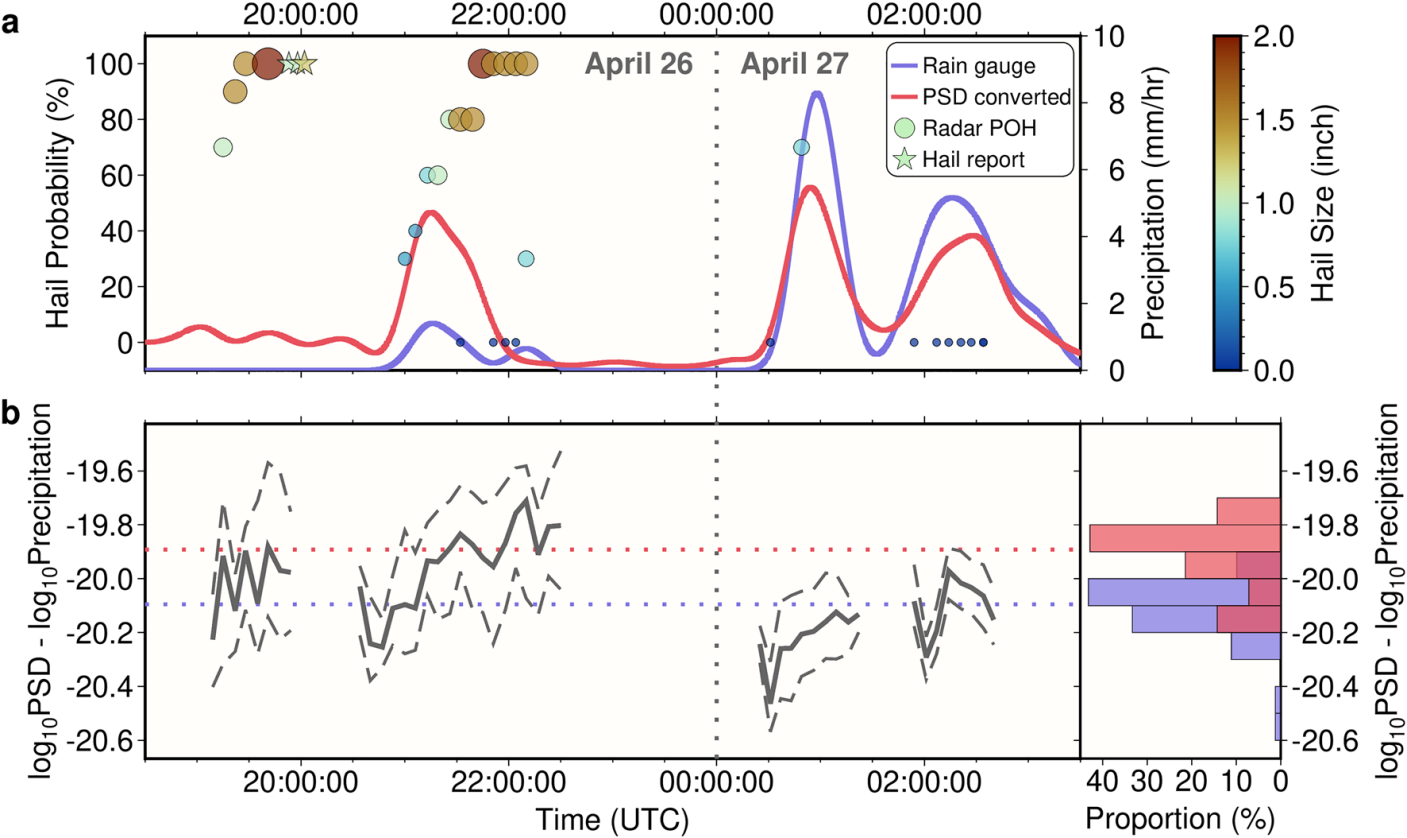
The relationship between seismic PSD and hailfall. (**a**) Similar to Fig. 3c, the blue line shows the smoothed rain gauge precipitation rate for the event on 26 April 2016, and the red line shows the smoothed seismic PSD converted precipitation rate using the relationship in Fig. 3e. A clear overestimation appears at around 21:15 UTC. Circles show the probability of hail of any size (POH) estimated from the ground-based weather radar, and both their sizes and colors show the maximum expected hail size (MEHS). These hail related parameters are only plotted when their corresponding storm cell location is less than 0.5 km from the closest place with rainfall (over 0.3 mm h^−1^ precipitation rate indicated by both the radar and the seismic PSD relationship in Fig. 3e). Times with multiple nearby storm cells may show multiple circles. Stars show human hail reports (treated as POH 100%) that are less than 10 km from the closest seismic station, with colors showing the reported hail size. The storm cell before 20:00 UTC did not pass the rain gauge (Movie S5). (**b**) The left panel shows the PSD-PR difference when the total precipitating areas are larger than 20 km^2^. Solid line shows the median value for the precipitating area, while dashed lines show 25th and 75th percentiles. The right panel shows histograms for the median of this PSD-PR difference (solid grey lines in the left panel) for all events (Fig. S5). Red bars are collected at times when POH is greater than 80%, and blues bars are collected when POH is zero. Median values for these two histograms are plotted as dotted lines in the left panel.

Such PSD-PR differences are compared with probability of hail (POH) and maximum expected hail size (MEHS) estimated from the ground-based radar (Fig. 5 and Fig. S5). These radar hail parameters are generated by the WSR-88D radar’s Hail Detection Algorithm based on large reflectivity values above the freezing level, and are available for individual storm cells, with storm cell center locations also given^20^. Here, we only consider hail parameters from storm cells whose centers are less than 500 m away from precipitating locations (where both radar and seismic PSD indicate a precipitation rate higher than 0.3 mm h^−1^). Human reports are also considered when their minimum distance to the array is less than 10 km (stars in Fig. 5, Fig. S5). Figure 5 shows that a larger PSD-PR difference occurs when POH and MEHS are greater. For example, from 20:30 to 22:00 UTC on 26 April 2016, such differences increase along with increases in POH and MEHS (Fig. 5), consistent with the abnormally high seismic PSD converted precipitation rate (Fig. 5a). We noted that POH and MEHS appear later than precipitation recorded by the rain gauge (Fig. 5a), likely caused by the difference in location between the radar reported storm cell and the rain gauge, given that the PSD-PR difference does not show a time lag (Fig. 5b). Overall, when POH is higher than 80%, the PSD-PR difference is systematically higher than the case when POH is zero (Fig. 5b), suggesting the potential to detect hail using a seismic array. Compared with existing probabilistic hail indices from weather radar, our seismic approach is likely to map the spatial distribution of hailfall more accurately, and could serve as an automatic surface measurement in conjunction with other approaches (e.g., hailpads).

## Discussion

Though our seismic-derived precipitation estimates infer precipitation indirectly through droplet impact, they show strong potential to complement existing monitoring approaches by leveraging five distinct advantages: (1) extremely high temporal resolution (1–10 s); (2) very high spatial resolution (~ 500 m); (3) sensitivity to raindrop or hailstone sizes; (4) influence only from precipitation reaching the ground; and (5) wider spatial sampling extent compared to rain gauges.

The time resolution of seismic precipitation signals is higher than traditional approaches to precipitation monitoring. For instance, the operational weather radar used in this study offers instantaneous precipitation rate not more frequent than every 5 min due to its scanning strategy (Movies S1–S9), and sometimes provides precipitation estimates inconsistent with those from the rain gauge and rain-gauge-calibrated seismic PSD (Figs. 2 and 3d). This discrepancy might occur because radar precipitation accumulation estimates without gauge-radar bias are only available hourly^16^. Meanwhile, tipping-bucket rain gauge measurements are often not continuous (e.g., Fig. 3d). Satellite precipitation products also usually have a lower time resolution from minutes to hours. In contrast, the seismic signals are analyzed at a much higher frequency (100–200 Hz), resulting in a temporal resolution on the order of seconds in this study.

Seismic monitoring also offers higher spatial resolution. Compared with operational weather radar, seismic surface measurements reveal narrower precipitation areas (Fig. 4, Fig. S3, Movies S1–S9). Moreover, although radar products offer precipitation rates at high nominal spatial resolution (e.g., Fig. S3c,f), raw radar data often contains abrupt changes between neighboring locations partly due to oversampling during data retrieval, and the revealed precipitation region from these unsmoothed data is in general still broader than the surface seismic measurement (Fig. S3). The spatial resolution of precipitation estimates from space-borne radars is even lower, on the order of kilometers^36^. If tipping-bucket rain gauges were deployed as part of a dense array, they would have an apparent high spatial resolution as well, but their effective resolution would be restricted by their temporal resolution with respect to a moving precipitating feature. For a rain gauge with an instrument sensitivity of 0.2 mm, if the precipitation rate is 1 mm h^−1^, a rain gauge can only record precipitation every 12 min, which would translate into a spatial resolution of 4 km if the precipitating feature travelled at a speed of 20 km h^−1^.

Another unique feature of the seismic measurement is its sensitivity to particle sizes (Eq. 2). Currently, to directly measure raindrop or hailstone sizes, a high-cost disdrometer^37^ or a hand-operated hailpad^12^ is required. For actual hail monitoring, common practices depend on human reports, a labor-intensive hailpad network^13, 14^, or hail information retrieved from weather radars. Estimates from radar depend on calibration from the relatively limited number of hailpad measurements^38^, often mismatch the location from hail reports^39^, and do not provide accurate spatial information inside a storm cell. In contrast, the seismic PSD itself strongly depends on the hydrometeor (hailstone) kinetic energy^21–23^ (Eq. 2), which is evident when PSDs are compared with rain gauge precipitation rates (Fig. 5a). The computed PSD-PR differences are broadly consistent with radar-based hail indices (Fig. 5b), showing the capability of hail monitoring when independent seismic and precipitation measurements are available. In addition, the PSD-PR difference could potentially resolve the hailfall spatial distribution. This hail detection capability still requires further investigation and calibration since here we only analyzed a few events with insufficient ground truth. Thus, the hail-related analysis only illustrates a potential use of the PSD-PR difference. Such a potential hail detection capability points to the usefulness of designing collaborative observational programs between the seismology and meteorology communities. Specifically, during future deployments of high-density seismic nodal arrays, a coordinated meteorological field campaign with the deployment of disdrometers and hailpad arrays would help unveil the seismic characteristics of hailstones at different sizes and move toward a multidisciplinary real-time product of hail detection and characterization.

Moreover, seismic precipitation monitoring also benefits from its surface measurement nature and a larger spatial extent of sampling. Compared with ground-based and space-borne radars, which remotely sense hydrometeors in the air, seismic signals are generated by actual raindrops hitting the ground^21, 22^. Particularly for the potential usage in hail detection, a ground-based radar is prone to bias aloft due to strong attenuation during a convective storm^40^, and it can only produce hail data in a probabilistic sense which, in contrast, are not problems for the seismic surface measurement. Meanwhile, assuming precipitation seismic signals are mainly Rayleigh waves^29, 41^, the seismic PSD is sensitive to combined impacts from raindrops within ~ 5–25 m^21^, a much wider areal extent than sampled by rain gauges, ensuring a continuous precipitation measurement and avoiding random errors due to infrequent raindrop sampling over a small area.

Further improvements could be made to the seismic monitoring approach. (1) The Earth structure response has a different frequency dependence for various soil types and burial depths^22, 23^, so it could be better corrected in a frequency-dependent way, which could be easily adjusted based on the method in this study. (2) Thunder signals^42^ are not fully removed during denoising (e.g., Movie S1). (3) While rain signals are found up to 450 Hz^22, 43^, LASSO seismic stations cannot resolve signals over 250 Hz, and its data quality is problematic above 200 Hz (Fig. 2b), suggesting better instrumentation would improve the monitoring. (4) The precipitation process itself could influence the seismic record and is worth further investigation. Infiltrated water could potentially saturate the soil and change the nearby structural response, and the surface rainwater could form a water film that reduces the impact intensity. However, the overall low relative structure response uncertainties (Fig. S1) indicate that those effects are likely secondary. (5) The size distribution and fall speed of hailstones likely differ from those of raindrops. Hence, more experiments are required to better understand seismic hail signals. (6) Theoretically, at higher frequencies, the precipitation seismic PSD would be greater but only from raindrops falling within shorter distances to the station^21^. Thus, for different precipitation events, the optimal frequency band could be different, e.g., regular precipitation events could benefit from higher frequencies due to potentially easier PSD discrimination, but sparsely distributed hail may require lower frequencies for the measurement to be robust. (7) Though the unit price of current nodal seismometers is not high, the total cost is still considerable for an array with a large number of nodes. Since these seismic nodes are designed to sample seismic waves at much lower frequencies than are used in this study, alternative sensors, like low-cost seismometers or accelerometers, may be cost effective and perform well for precipitation monitoring. Further investigation of different sensors could help bring this method into practical use.

With these special characteristics of seismic monitoring, though only deployed for one month, interesting meteorological phenomena were revealed. For example, a discrete supercell thunderstorm tracked northeastward over the domain with a relatively narrow, yet intense swath of high-precipitation rates and associated accumulation between around 21:30 UTC and 22:30 UTC on 9 May 2016 (Movie S9). The improved spatial and temporal resolution of the surface seismic monitoring measurements are exemplified in the comparison between the seismic converted one-hour precipitation accumulation and the radar one-hour precipitation accumulation. The seismic converted one-hour precipitation accumulation shows a more detailed and higher precipitation accumulation swath compared to the radar one-hour precipitation accumulation swath for the supercell thunderstorm (Movie S9). Other similar examples include between around 16:30 UTC and 17:00 UTC on 29 April 2016 (Movie S6) and between around 16:00 UTC and 16:30 UTC on 8 May 2016 (Movie S7). This pattern not only was present with isolated storm modes, but also was apparent with linear bands of thunderstorms, such as between around 21:00 UTC and 22:30 UTC on 26 April 2016 (Movie S5).

Together, with these experimental monitoring practices, we found seismic array analysis to be a strong complement to existing precipitation monitoring approaches, due to its high spatial and temporal resolution, and the sensitivity to raindrop or hailstone sizes. Thus, with further investigation, this seismic approach could potentially be used to extend and improve hazard mitigation systems for hailstorms or intense thunderstorms.

## Methods

### Influence of the raindrop size distribution on seismic signals

Generalized from the exponential distribution in an early study^44^, the raindrop size distribution (DSD) is often parameterized as a normalized Gamma distribution^45^:

4 $$N(D) = \frac{{N_{0} }}{{D_{0} }}\left( {\frac{D}{{D_{0} }}} \right)^{\mu } e^{{ - \left( {\mu + 4} \right)\frac{D}{{D_{0} }}}} ,$$

where *D* is the droplet equivolume spherical diameter, and *N*_0_, *D*_0_, and *μ* are all constants. *N*_0_ characterizes the number of raindrops; *D*_0_ characterizes the average size of raindrops; and *μ* controls the shape of the DSD. An example of this DSD is shown in Fig. S6a, and Eq. (4) can well fit observations. We assume all droplets have reached their terminal fall speed, and this fall speed only depends on the droplet size (*D*) and some known constants. One formulation^46^ involves the density of liquid water and air (*ρ*_*w*_ and *ρ*_*a*_) and a dimensionless drag coefficient (*c*), with the square of the fall speed, *v*^2^, being proportional to *D*:

5 $$v^{2} = \frac{4}{3}gD\frac{{\rho_{w} - \rho_{a} }}{{\rho_{a} c}},$$

where *g* is the gravitational acceleration. Some other formulations also have a polynomial relationship between *v* and *D*^47, 48^, but one can show that these assumptions lead to similar conclusions. In this case, similar to Ref.^21^, the droplet average |*NF*^2^| appearing in Eq. (2) should be written as an integral with respect to the spherical diameter:

6 $$\left| {NF^{2} } \right| = \int_{0}^{\infty } {N(D)m^{2} (D)v^{2} (D)dD} .$$

With the mass of raindrops given by:

7 $$m = \frac{1}{6}\pi \rho_{w} D^{3} ,$$

then, combining Eqs. (6) and (7) with Eq. (5):

8 $$\left| {NF^{2} } \right| = \left( {\frac{1}{6}\pi \rho_{w} } \right)^{2} \cdot \frac{4}{3}g\frac{{\rho_{w} - \rho_{a} }}{{\rho_{a} c}}\frac{\Gamma (\mu + 8)}{{\left( {\mu + 4} \right)^{\mu + 8} }}N_{0} D_{0}^{2} = \frac{\Gamma (\mu + 8)}{{\left( {\mu + 4} \right)^{\mu + 8} }}N_{0} m_{0}^{2} v_{0}^{2} ,$$

where Γ is the gamma function, and *m*_0_ and *v*_0_ are the mass and the fall speed of a droplet of size *D*_0_, respectively. Similarly, the precipitation rate is characterized by:

9 $$PR = \frac{1}{{\rho_{w} }}\int_{0}^{\infty } {N(D)m(D)dD = \frac{\Gamma (\mu + 4)}{{\rho_{w} (\mu + 4)^{\mu + 4} }}N_{0} m_{0} } ,$$

and the average raindrop kinetic energy would be:

10 $$E = \frac{{\int_{0}^{\infty } {N(D)\frac{1}{2}m(D)v^{2} (D)dD} }}{{\int_{0}^{\infty } {N(D)dD} }} = \frac{\Gamma (\mu + 5)}{{2(\mu + 4)^{4} \Gamma (\mu + 1)}}m_{0} v_{0}^{2} .$$

Therefore, combining Eqs. (9) and (10) with Eq. (8), we obtain:

11 $$\left| {NF^{2} } \right| = \frac{\Gamma (\mu + 8)\Gamma (\mu + 1)}{{\Gamma (\mu + 5)\Gamma (\mu + 4)}}2\rho_{w} \cdot PR \cdot E.$$

This relationship suggests that the seismic PSD is directly dependent on the precipitation rate and raindrop kinetic energy.

Since Eq. (11) shows the PSD is also dependent on the shape of the DSD (*μ*), we examined its potential influence based on raindrop sizes recorded by an impact disdrometer located ~ 30 km away. Between April 2016 and November 2021, for any day with precipitation accumulation over 2 mm, we first fit the observed DSD with Eq. (4) by solving for the unknown parameters, *μ*, *N*_0_, and *D*_0_ (e.g., Fig. S6a). This optimization is done by expressing Eq. (4) in log-scale,

12 $$\log N = \mu \log D - \frac{\mu + 4}{{D_{0} }}D + \left[ {\log N_{0} - (\mu + 1)D_{0} } \right].$$

Through Eq. (12), we can estimate the parameters needed from a bivariate (log*D* and *D*) linear regression using the ordinary least square method to get the three parameters, with the slope for log*D* being the shape parameter *μ*. Optimized *μ* for the 257 precipitation days is between 0.5 and 3 the majority of the time (Fig. S6a).

We then evaluate the potential influence of varying *μ* on the seismic PSD. With the disdrometer providing *N*(*D*), *m*(*D*), and *v*(*D*), the average raindrop kinetic energy (*E*) is calculated based on the first half of Eq. (10). Similarly, we also quantified |*NF*^2^| and *PR* (Eqs. 6 and 9), so that the PSD-PR difference can be directly estimated based on disdrometer observed data. The observed PSD-PR difference is then compared with both the observed average raindrop kinetic energy and *μ* (Fig. S6b). We found that although *μ* can influence this difference (Eq. 11), PSD-PR highly correlates with *E* instead of *μ*, which suggests the potential to retrieve raindrop kinetic energy from the PSD-PR difference.

Meanwhile, the parameter *μ* does not need to vary much in order to fit the observed DSD. As shown in Fig. S6a, an example of the observed DSD can be successfully characterized when specifying different values of *μ*. In addition, the overall relationship between *E* and the PSD-PR difference in Fig. S6b is well reproduced using a constant *μ* of 2. We also systematically evaluate the coefficient of determination for fitting (*r*^2^) for all 257 days, in the context of optimized or prescribed *μ* (Fig. S6c). We found that although the highest *r*^2^ is achieved when *μ* is optimized, *r*^2^ is still high using a constant *μ* of 2, with 63% of *r*^2^ over 0.98 and 90% of *r*^2^ over 0.95 (Fig. S6a). This finding further substantiates our treatment of the seismic PSD as a function of the precipitation rate and the raindrop kinetic energy.

### Seismic power spectral density calculation and noise removal

To calculate seismic PSDs, we used Welch’s method^28^. In this method, at every second, we extract seismic records that are ± 5 s around it. This 10 s section is then divided into 200 windows, each with a length of 0.1 s and overlaps the neighboring window by 50%. For each window, the PSD is calculated for 10–250 Hz, and the average of these 200 windows is used to represent the seismic PSD at each time. Therefore, the effective temporal resolution in this study is between 1 and 10 s. We used a long time section because seismic PSD data below 100 Hz were also used for analysis. However, since precipitation signals mainly appear above 100 Hz (Fig. 2b), the seismic PSD at 100–250 Hz could be confidently estimated using a short 1 s section with 0.02 s long windows, which would allow for a true 1 s temporal resolution.

After calculating the seismic PSD, anthropogenic noise is removed. During denoising, four frequency bands are used, the overall band 50–200 Hz (*PSD*_50–200_); the first low frequency band 15–35 Hz (*PSD*_15–35_); the second low frequency band 50–70 Hz (*PSD*_50–70_); and the high frequency band 170–190 Hz (*PSD*_170–190_). The full frequency band is used to identify strong pulses that are restricted in time. The two low frequency bands were chosen as the noise bands semi-empirically by inspecting the seismic spectra and evaluating the related denoising performance, and they are close to the two previously detected noise bands^23^ at 4–25 Hz (human activities) and 40–80 Hz (potentially related to thermoelastic and meteorological conditions).

In practice, we first found all potential times for noise if one of the following 6 criteria is met: (1) $PSD_{50 - 200} \times PSD_{50 - 70} /PSD_{170 - 190}$ is over three times its root mean square value for the whole event (rms); here, a large *PSD*_50–200_ identifies times with a large overall amplitude, while a large *PSD*_50–70_/*PSD*_170–190_ means the noise level at 50–70 Hz is higher than normal, so a combined large value indicates the PSD is large and could be caused by an elevated noise level. (2) $PSD_{50 - 200} \times PSD_{15 - 35} /PSD_{170 - 190}$ is over three times its rms; the logic behind this criterion is similar to (1) but is for the noise frequency band at 15–35 Hz. (3) $PSD_{50 - 200} /{\text{mean}}_{{100{\text{s}}}} (PSD_{50 - 200} )$ is over three times its rms, where mean_100s_ stands for taking the average *PSD* around ± 50 s (a 100 s section); this criterion identifies anomalous short pulses with an elevated seismic PSD compared to the averaged value. (4) $PSD_{50 - 200}^{2} /{\text{mean}}_{100s} (PSD_{50 - 200} )$ is over three times its rms; this criterion serves for a similar purpose to (3), and the square in the numerator is to exaggerate pulses with high amplitudes. (5) ${{\left[ {{\text{mean}}_{{10{\text{s}}}} (PSD_{50 - 200} )} \right]^{2} } \mathord{\left/ {\vphantom {{\left[ {{\text{mean}}_{{10{\text{s}}}} (PSD_{50 - 200} )} \right]^{2} } {{\text{mean}}_{{40{\text{s}}}} (PSD_{50 - 200} )}}} \right. \kern-0pt} {{\text{mean}}_{{40{\text{s}}}} (PSD_{50 - 200} )}}$ is over three times its rms; this criterion is also to identify anomalous pulses, but here the numerator is averaged around ± 5 s for longer pulses. (6) $PSD_{50 - 200} /{\text{median}}_{{60{\text{s}}}} (PSD_{50 - 200} )$ is over three times its rms, where median_60s_ stands for the median *PSD* around ± 30 s; this criterion is for spotting short pulses as well, but here the seismic PSD is compared with a less smoothed reference value (60 s vs. 100 s in criterion 3). Overall, the first two criteria are designed to identify times with strong amplitudes at noise frequencies, and the other four are designed to identify pulses with high amplitudes.

After identifying potential noise, we attempted to identify time windows for noise and remove them. We first obtained time windows with minimum lengths that could contain all these noisy times. Then, when two neighboring windows are both identified to have noise, if the minimum *PSD*_50–200_ in their interval is higher than one-third of the maximum *PSD*_50–200_ of either window, the two windows are concatenated as one. After that, in each window, we searched beyond its two boundaries to find the first time on either side that the *PSD*_50–200_ is below one-third of the maximum *PSD*_50–200_ within the window as the final boundaries of the window (to include tails of the noise in the window). Since the targeted noise is restricted in duration, only windows with short durations are considered as real noise windows. In practice, if the maximum *PSD*_50–200_ is over 10^−16^ m^2^/Hz, the window would be considered a noise window if its length is less than 24 s; otherwise, windows less than 16 s duration are counted as noise. With the noise windows determined, we discarded the seismic PSD at these times, and interpolated the PSD with 10 s from each side of the noise window to fill in the discarded part.

### Earth structure response correction

To systematically analyze seismic PSDs from all stations, common precipitation windows for neighboring stations were first identified. Two stations are considered neighboring stations if their distance is within 1.5 km. Common precipitation windows were determined based on their average seismic PSD between 100 and 200 Hz (e.g., Fig. 2d). Smoothed seismic PSDs at all time points were first obtained by averaging the original PSD around a time window ± 15 s of each time point, and a noise level is defined as the root mean square of the smoothed seismic PSD before and after the precipitation event. We then find all windows with seismic PSD greater than three times the noise level and longer than 90 s. We also required these windows to have a maximum PSD higher than six times the noise level, and the average PSD within the window greater than 70% of the average PSD when the window has both sides extended by 2 min, to ensure that the window not only contains high amplitudes but also those high amplitudes are not for a trough between two large peaks. Windows with lengths over 20 min are divided into multiple 20-min windows. Each window then has its two sides extended by 40 s and tapered to zero. The signal-to-noise ratio (*snr*) for each window is defined as the maximum PSD within the window divided by the noise level.

With these precipitation windows, the seismic PSD differences for station pairs (Eq. 3) are measured. For each pair, we first calculated the cross-correlation between their seismic PSD time series (*psd*). If the maximum value of the cross-correlation (*CC*_max_) appears within 3.5 min of the zero time, suggesting precipitation happens at similar times for the two events, we calculated their PSD difference by:

13 $$\log \frac{{PSD_{i} }}{{PSD_{k} }} = \log \frac{{CC_{max} (psd_{i} ,psd_{k} )}}{{CC_{0} (psd_{k} ,psd_{k} )}},$$

where *CC*_0_ is the cross-correlation value at zero time, and *i*, *k* are indices for two stations. The quality of the cross-correlation is characterized by the correlation coefficient (*cc*) as:

14 $$cc = \frac{{CC_{max} (psd_{i} ,psd_{k} )}}{{\sqrt {CC_{0} (psd_{i} ,psd_{i} ) \times CC_{0} (psd_{k} ,psd_{k} )} }}.$$

Then, we defined a weighting function as:

15 $$w(x,a,b) = \left\{ {\begin{array}{*{20}l} {0,} \hfill & {x \le a} \hfill \\ {0.5 - 0.5\cos (\pi \frac{x - a}{{b - a}}),} \hfill & {a < x < b} \hfill \\ {1,} \hfill & {x \ge b} \hfill \\ \end{array} } \right.,$$

and with these, the weight for each station pair PSD difference measurement is defined as:

16 $${\mathfrak{w}}_{i,k} = w(cc,0.55,0.95) \times w(snr_{i} ,4,10) \times w(snr_{k} ,4,10) \times w(T,0,10),$$

where *T* is the length of the window in min. After obtaining weights for each measurement window using Eq. (16), we calculated the weighted average of the PSD difference ($\log \left( {{{PSD_{i} } \mathord{\left/ {\vphantom {{PSD_{i} } {PSD_{k} }}} \right. \kern-0pt} {PSD_{k} }}} \right)$) for each individual measurement window. Subsequently, the source effect in Eq. (3) can be considered random for different measurement windows and is eliminated, and thus such averaged $\log \left( {{{PSD_{i} } \mathord{\left/ {\vphantom {{PSD_{i} } {PSD_{k} }}} \right. \kern-0pt} {PSD_{k} }}} \right)$ represents the overall PSD difference between the station pair (*r*_*i,k*_). In practice, after obtaining *r*_*i,k*_ and the standard deviation (*std*_*i,k*_) for those measurements, we eliminate those windows with measured $\log \left( {{{PSD_{i} } \mathord{\left/ {\vphantom {{PSD_{i} } {PSD_{k} }}} \right. \kern-0pt} {PSD_{k} }}} \right)$ not within 2.5 times the *std*_*i,k*_ around the *r*_*i,k*_, and this operation is performed iteratively until all windows are within this range or the number of windows is less than 10. For the following analyses, to obtain the relative Earth structure response, we used a weight $W_{i,k} = \sum\nolimits_{windows} {{\mathfrak{w}}_{i,k} }$ to characterize the overall quality of measurements for each station pair.

The relative Earth structure response (*R*) with respect to a reference station is then calculated based on Newton’s method^32^. In this study, the optimal Earth structure response for stations was found by minimizing the following cost function:

17 $$J({\mathbf{R}}) = \sum\limits_{i,k} {W_{i,k} \frac{{\left( {{\mathcal{R}}_{i} - {\mathcal{R}}_{k} - r_{i,k} } \right)^{2} }}{{2std_{i,k}^{2} }}} ,$$

where ${\mathcal{R}}_{i}$ stands for the log-scale relative structure response (log*R*_*i*_), and is the *i*th component of the vector **R**. The *m*th component of the gradient (**g**) of the cost function (with respect to ${\mathcal{R}}_{m}$) is then expressed as:

18 $$g_{m} = \sum\limits_{i,k} {W_{i,k} \frac{{\left( {{\mathcal{R}}_{i} - {\mathcal{R}}_{k} - r_{i,k} } \right)\left( {\delta_{im} - \delta_{km} } \right)}}{{std_{i,k}^{2} }}} ,$$

where δ here is the Kronecker delta. Then the (*m*th,* n*th) element of the Hessian matrix (**H**), i.e., the gradient of **g**, can be expressed as:

19 $$H_{mn} = \sum\limits_{i,k} {\frac{{W_{i,k} }}{{std_{i,k}^{2} }}\left( {\delta_{im} - \delta_{km} } \right)\left( {\delta_{in} - \delta_{kn} } \right)} .$$

With the Hessian and gradient, based on Newton’s method^32^, the optimization can be iteratively updated from the *l*th to the *l* + 1th iteration by:

20 $${\mathbf{R}}_{l + 1} = {\mathbf{R}}_{l} - {\mathbf{H}}_{l}^{ - 1} {\mathbf{g}}_{l} .$$

However, because the Hessian in this problem is explicit and not dependent on **R**, the cost function is quadratic, and for any assumed starting **R** the same final optimal **R** can be achieved by updating Eq. (20) once, i.e., the problem does not require multiple iterations. Together with the optimal relative Earth structure response **R** (Fig. S1a) obtained through Eq. (20), the uncertainty for** R** is also obtained. Because the covariance matrix for **R** is just the inverse of the Hessian matrix in Eq. (19)^33^, square roots of diagonal elements in this covariance matrix (**H**^−1^) are standard deviations (σ) of the relative Earth structure response (log*R*) for all stations (Fig. S1b). Station 340 (Fig. S1) is set as the reference station because the summed standard deviation from all other stations is minimal.

### Obtaining the seismic PSD at each location through spatial averaging

After obtaining seismic PSDs at different stations, and having their relative Earth structure responses removed, we used weighted spatial averaging to obtain the seismic PSD for each location within the study area. The weight for one precipitation event at a specific station is defined as:

21 $${\mathcal{W}} = \left[ {1 - w(\sigma ,0.03,0.08)} \right] \times w(noise,19.3,20.2) \times \left[ {1 - w(d,0.1,1.7)} \right],$$

where *w* is the weighting function in Eq. (15); σ is the standard deviation for the log-scale relative Earth structure response ${\mathcal{R}}$; *d* is the distance from the station to the location of interest in km; and *noise* is the noise level we estimated for this precipitation event at the station. To estimate the noise level, we first obtained the log_10_ scale of the time series for the average seismic PSD between 100 and 200 Hz, which is then smoothed by taking the average around ± 5 s. The noise level (*noise*) is defined by the root mean square of this smoothed time series at times before and after precipitation. Because log_10_PSD is always negative, after taking the root mean square, the lower the *noise* value, the stronger the noise is. With the weight in Eq. (21), the time series after being transformed into log-scale (*psdl*) at a random location is obtained by:

22 $$psdl = \frac{{\sum\nolimits_{i} {{\mathcal{W}}_{i} {\mkern 1mu} psdl_{i} } }}{{\sum\nolimits_{i} {{\mathcal{W}}_{i} } }},$$

where *i* is the index for the station. The total weight at the location $\sum\nolimits_{i} {{\mathcal{W}}_{i} }$ is used to characterize the reliability of the averaging. For maps shown in this study (e.g., Fig. 4), only places with total weight higher than 1.5 are considered to have a sufficient amount of data and are analyzed.

### Converting seismic PSD to precipitation rate

To convert seismic PSDs for individual sub-events (Fig. 3) to precipitation rate based on Eq. (2), first we rewrite the relationship in log-scale as:

23 $$\log PR = \log PSD - \log E - \log 2\rho_{w} S.$$

Here, the last part of the right-hand side is a constant among stations after removing the relative Earth structure responses, and if *E* depends on the precipitation rate, the relationship is characterized by:

24 $$\log PR = p\log PSD + q,$$

where *p* and *q* are the slope and intercept of a linear model to be determined. These two values were obtained through the ordinary least-squares method, and all-time steps with both smoothed precipitation rates (e.g., Fig. 3c) over 1 mm h^−1^ and smoothed seismic PSD (e.g., Fig. 3b) over three times the mean PSD noise level among neighboring stations were used in the linear regression. The noise level was defined when forming the weighting function (Eq. 21) for spatial averaging.

To obtain the overall linear relationship in Fig. 3e, the same relationship in Eq. (24) and the ordinary least-square method was applied. However, different sub-events have different durations and both the seismic and rain gauge records are continuous. Thus, to weight all sub-events equally, we discretized each sub-event to the same number of time points using the bootstrap method. At each iteration, 150 time points were randomly picked from each sub-event (except the two abnormal ones, the first sub-event on 26 April 2016 and the event on 18 April 2016, shown as dashed lines in Fig. 3e), and the seismic PSD was fitted to the precipitation rate for these times using the ordinary least-square method to obtain *p* and *q* in Eq. (24). We ran 3000 iterations in total, and the average *p* and *q* were used to represent the overall relationship in Fig. 3e. The 95% prediction interval that characterizes the spread of the data (Fig. 3e) was also obtained by averaging prediction intervals obtained at those iterations.

## Supplementary Information

Supplementary Information 1.

Supplementary Video 1.

Supplementary Video 2.

Supplementary Video 3.

Supplementary Video 4.

Supplementary Video 5.

Supplementary Video 6.

Supplementary Video 7.

Supplementary Video 8.

Supplementary Video 9.

## Data Availability

All LASSO seismic data used in this study sampled between 17 April 2016 and 10 April 2016 were downloaded from the IRIS DMC (http://ds.iris.edu/ds/nodes/dmc/) with the network code of 2A. The data were requested for the precipitation period indicated by satellite monitoring for the region (e.g., Supplementary Movies), and 1 more hour was requested before and after that period to ensure the whole precipitation event is included. WSR-88D radar products at the Vance Air Force Base (KVNX), including Digital Accumulation Array (DAA), Digital Instantaneous Precipitation Rate (DPR), and Hail Index (HI), were obtained through NOAA’s Weather and Climate Toolkit (https://www.ncdc.noaa.gov/wct/index.php). Tipping-bucket rain gauge data and impact disdrometer data were from the E32 site and the C1 site, respectively, of the DOE ARM Southern Great Plains atmospheric observatory (https://www.arm.gov/capabilities/observatories/sgp/locations/). Hail reports were obtained from NOAA’s Storm Prediction Center Severe Weather Events Archive (https://www.spc.noaa.gov/exper/archive/events/). Satellite precipitation estimates shown in Movies were from the NOAA Climate Prediction Center Morphing Technique (https://www.cpc.ncep.noaa.gov/products/janowiak/cmorph_description.html).

## Code availability

All computer codes used for data processing, analysis, and plotting are available upon request.
