# Supplementary material for: High-resolution precipitation monitoring with a dense seismic nodal array
Source: Sci Rep. 2023 Jul 15;13:11450. doi: 10.1038/s41598-023-38008-w (PMC10349858; doi:10.1038/s41598-023-38008-w)
Supplement: Supplementary file 1 — Supplementary Information 1. [file 41598_2023_38008_MOESM1_ESM.pdf]

Supplementary Information

High-resolution Precipitation Monitoring with a Dense Seismic Nodal Array

Junlin Hua[1]*, Mengxi Wu[2], Jake P. Mulholland[3], J. David Neelin[4], Victor C. Tsai[5], Daniel T. Trugman[6]

1 Department of Geological Sciences, Jackson School of Geosciences, The University of Texas at Austin; Austin, TX, 78712 USA
2 Joint Institute for Regional Earth System Science and Engineering, University of California, Los Angeles, Los Angeles, CA, 90095 USA
3 Department of Atmospheric Sciences, John D. Odegard School of Aerospace Sciences, The University of North Dakota, Grand Forks, ND, 58202 USA
4 Department of Atmospheric and Oceanic Sciences, University of California, Los Angeles, Los Angeles, CA, 90095 USA
5 Department of Earth, Environmental and Planetary Sciences, Brown University, Providence, RI, 02912 USA
6 Nevada Seismological Laboratory, University of Nevada, Reno, Reno, NV, 89557 USA

Table of contents:

Supplementary Figure 1: Normalization of Earth structure responses
Supplementary Figure 2: Seismic precipitation measurements in comparison with rain gauge
Supplementary Figure 3: Precipitation spatial distribution from seismic array and weather radar
Supplementary Figure 4: Statistical comparison between precipitation spatial distribution estimation based on seismic PSD and weather radar
Supplementary Figure 5: The relationship between seismic power spectral density and potential hailfall
Supplementary Figure 6: The influence of raindrop size distribution
Supplementary Table 1: A summary of the nine observed precipitation events
Supplementary Table 2: The summary of symbols used in this study
Captions for Supplementary Movies

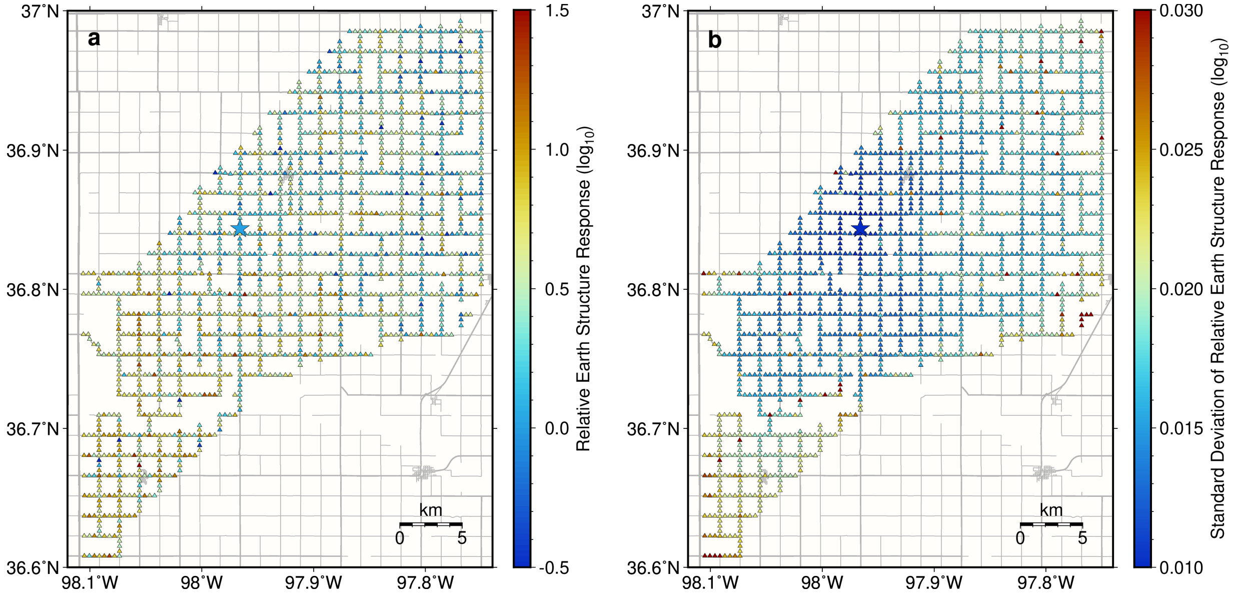

Supplementary Fig. 1 | Normalization of Earth structure responses. a. The same as Fig. 1, relative site responses for different stations with respect to the reference Station: 340 (star). **b.** The standard deviation for the obtained relative site response in **a**. The software used to create these maps is the Generic Mapping Tools, version 6.4.0[47] (https://www.generic-mapping-tools.org).

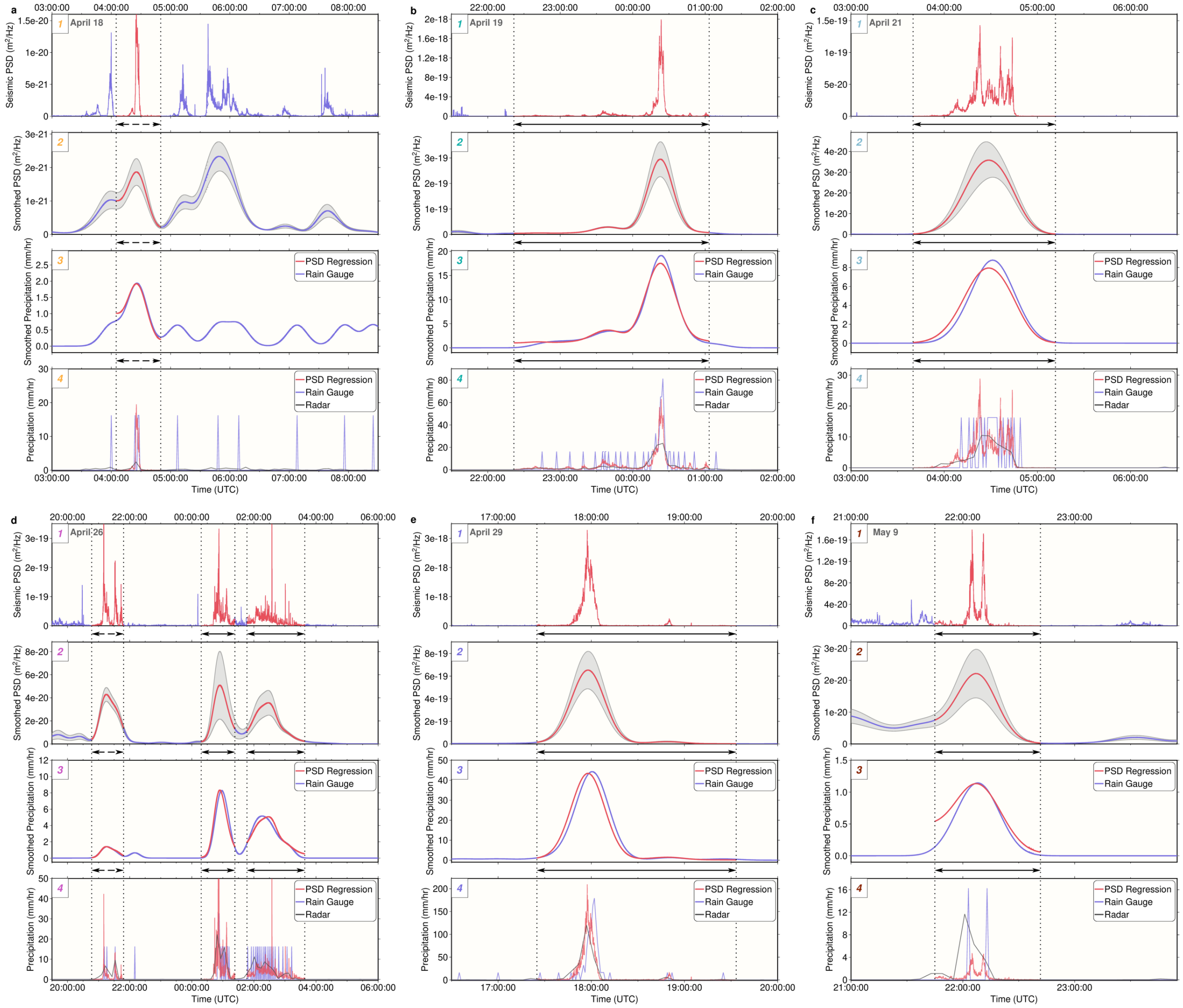

Supplementary Fig. 2 | Seismic precipitation measurements in comparison with rain gauge. a-f. correspond to events starting at the date indicated in the top left corner of their first sub-panel. **f** is for the second event on 9 May 2016. Events starting on 8 May 2016 (Event 7th, Table S1), and the first event on 9 May 2016 (Event 8th, Table S1) did not pass over the rain gauge location (Movie S7, Movie S8), so are not included in this figure. Four sub-panels in each panel correspond to the Figs. 3a-d, and colors of the panel numbers correspond to colors of lines in Fig. 3e.

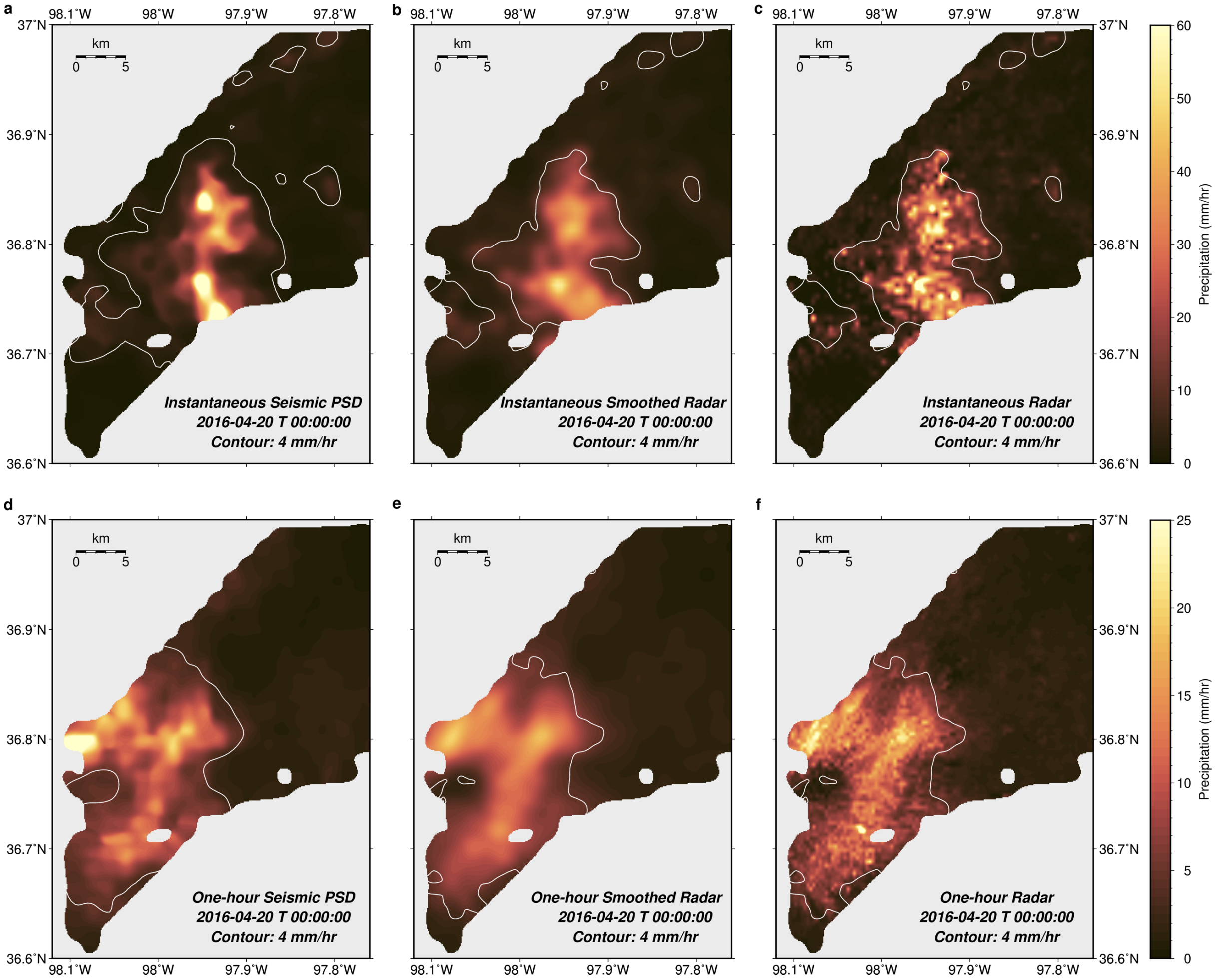

Supplementary Fig. 3 | Precipitation spatial distribution from seismic array and weather radar. a-c. Instantaneous precipitation rate at 00:00:00 UTC 20 April 2016 from converted seismic PSD (**a**, using the relationship in Fig. 3e); weather radar (**b**); weather radar without smoothing (**c**). White lines in **a** for the contour of 4 mm hr^{-1} in **b**, and white lines in **b** and **c** for the contour of 4 mm hr^{-1} in **a**. **d-f.** Similar to **a-c**, but for the averaged precipitation rate over one hour before the time, which is bias corrected with respect to the rain gauge for radar. Though without smoothing (**c** and **f**), the radar seems to provide a very high spatial resolution – those images are dominated by large oscillatory variations over short distances, and the unsmoothed radar precipitation region is still broader than the one from seismic array (**a** versus **c**). Gray areas are places with insufficient amount of data (criteria in Methods). The software used to create these maps is the Generic Mapping Tools, version 6.4.0[47] (https://www.generic-mapping-tools.org).

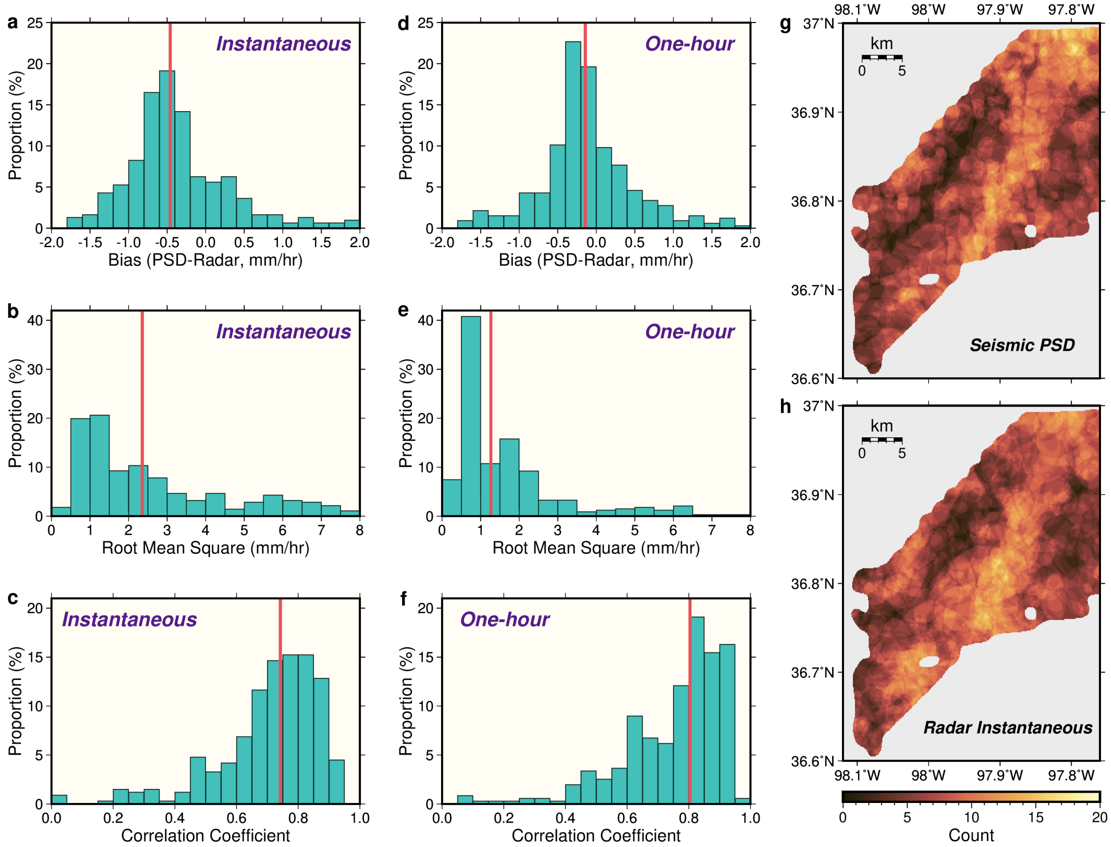

Supplementary Fig. 4 | Statistical comparison between precipitation spatial distribution estimation based on seismic PSD and weather radar. a-c are based on instantaneous seismic PSD and weather radar precipitation (e.g. Fig. S3a-b), while **d-f** are based on hourly averaged seismic PSD and weather radar precipitation (e.g. Fig. S3d-e). Seismic PSD precipitation estimations are based on the relationship in Fig. 3e. In these tests, each time point for which the radar provides a precipitation distribution map is viewed as an individual sample (e.g. Fig. 4, Fig. S3), and only time points with the average precipitation rate of the study region over 0.5 mm/hr are considered in the test. In **a** and **d**, to quantify the bias, at each time point, we calculated the spatially averaged difference in precipitation rate between the seismic PSD and weather radar-based distributions, and the histogram shows the distribution of such bias at different time points, while the red line shows the median value. **b** and **e**, show the corresponding root-mean-square difference between seismic PSD and weather radar-based estimations from calculations as in **a** and **d**. **c** and **f** show the corresponding distribution of the spatial correlation coefficient between seismic PSD and weather radar based precipitation distributions at different time points. **g** and **h** are heatmaps showing the number of times that seismic PSD and weather radar indicate higher than 25 mm/hr instantaneous precipitation rate (more likely to cause severe damage). These two plots combine data from all seven events, and each time when the radar updates its map, we identify locations exceeding 25 mm/hr. The software used to create this figure (including the two maps) is the Generic Mapping Tools, version 6.4.0[47] (https://www.generic-mapping-tools.org).

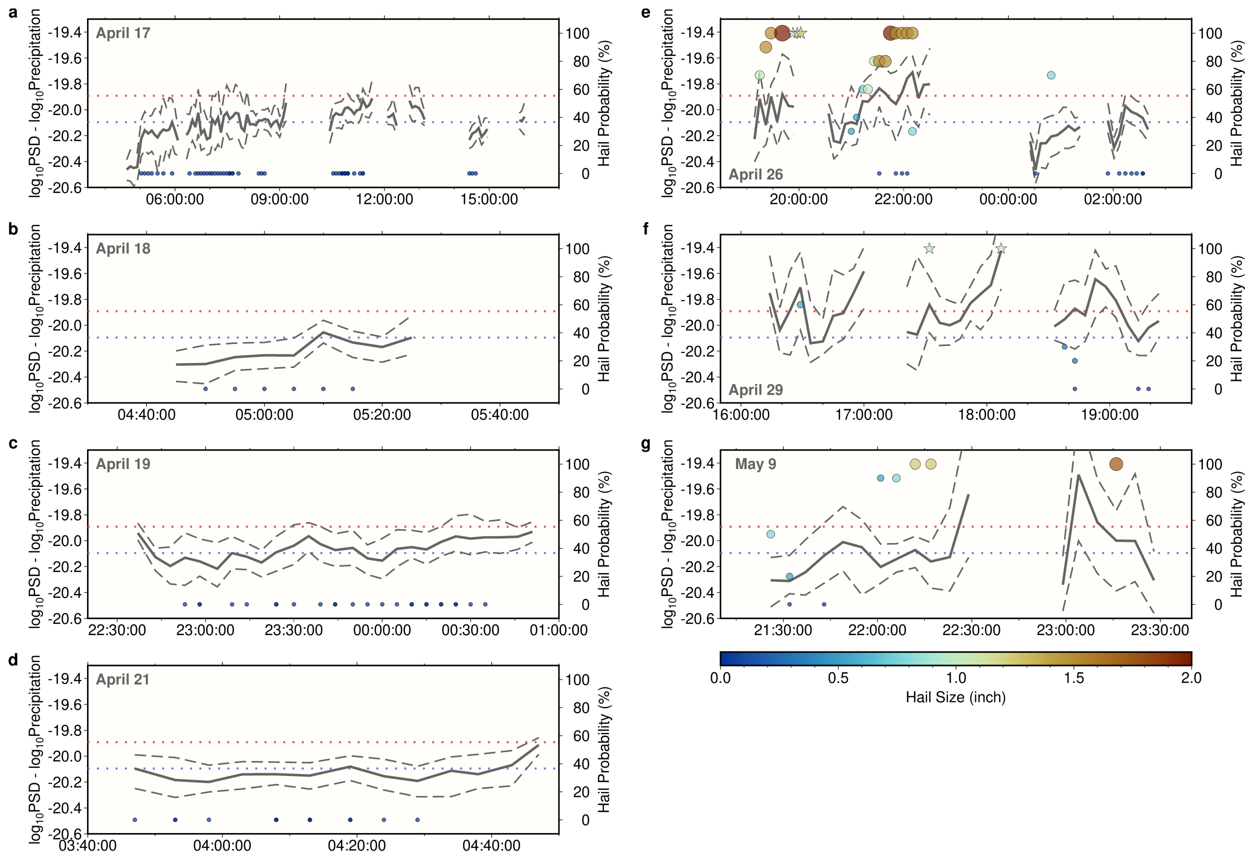

Supplementary Fig. 5 | The relationship between seismic power spectral density and potential hailfall. a-g. Seven precipitation events (time in UTC) with hail parameters provided by the radar (the same set of events as in Fig. 3 and Fig. S3, starting dates labelled at top left corners). Gray solid line shows the median value for the precipitating area, while dashed lines show 25th and 75th percentiles. Median values for the two histograms in Fig. 5b are plotted as dotted lines. As in Fig. 5a, circles show the probability of hail of any size (POH) estimated from the ground-based weather radar, and both their sizes and colors show the maximum expected hail size (MEHS). Stars show hail reports (treated as POH 100%) that are less than 10 km from the closest seismic station, with colors showing the reported hail size.

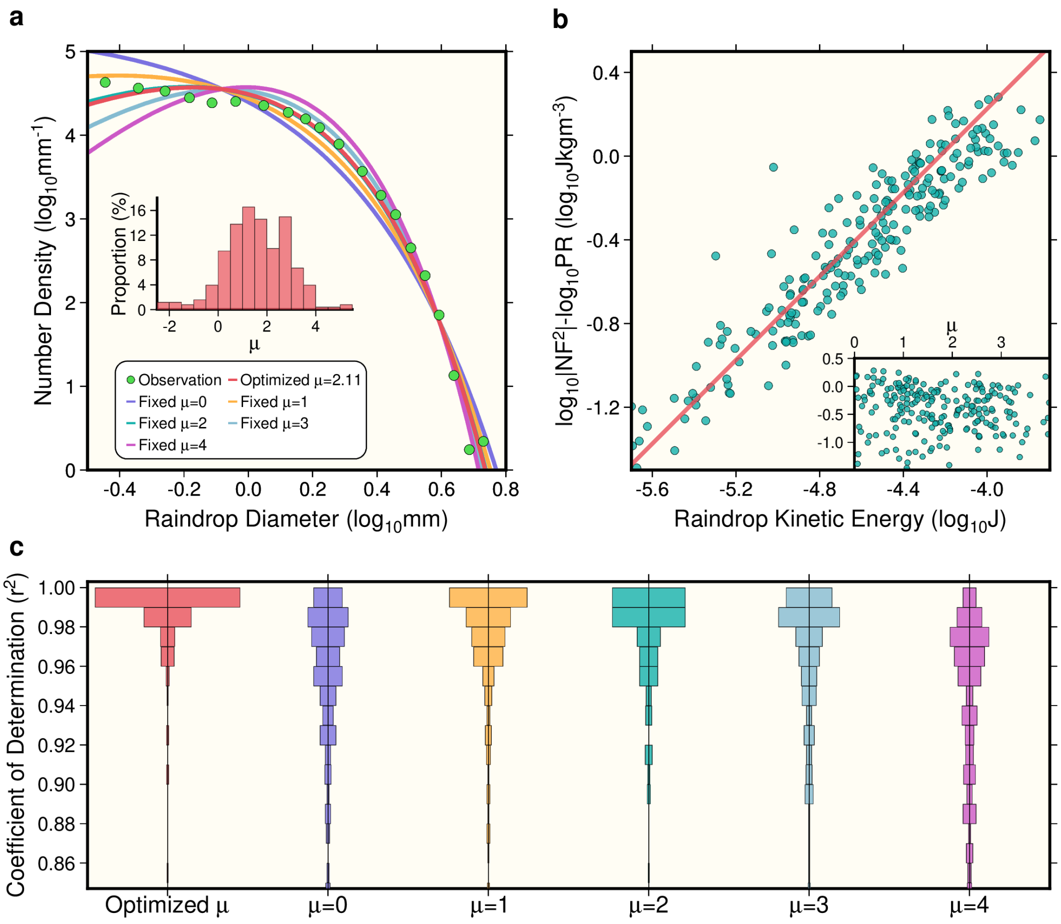

Supplementary Fig. 6 | The influence of raindrop size distribution. a. The raindrop size distribution from a disdrometer located ~30 km away from the study region on 4 April 2019. Dots are total counts of raindrops per diameter at that day in log-scale. Lines are fitted distribution based on Eq. (4). For the red line, μ, N_0, and D_0 are fitted together. For other lines, we prescribed μ from 0 to 4 and only solved N_0 and D_0. The histogram in **a** shows the distribution of fitted μ for days between April 2016 and November 2021 with accumulated rainfall greater than 2 mm (together 257 days). **b.** The relationship between raindrop kinetic energy (E) and the PSD-PR difference. Each dot corresponds to one day, and both axes are calculated based on provided N(D), m(D), v(D) from the disdrometer. The line, instead of based on disdrometer records, shows the theoretical relationship when μ is fixed at 2 (Eq. 11; y-axis: $2\rho_w[\Gamma(\mu+8)\ \Gamma(\mu+1)]/[\ \Gamma(\mu+5)\ \Gamma(\mu+4)]E$). The inset plot is based on the same set of data, but while y-axis is the same as the main plot, the x-axis is for fitted μ. This panel shows a much stronger relationship between the PSD-PR difference and the kinetic energy than that between the PSD-PR difference and μ. **c.** The distribution of coefficient of determination (commonly known as r^2) for the fitting of all the days. Six histograms are for cases where we fit μ, N_0, and D_0 together (optimized μ), or fix μ at different values and only solve N_0 and D_0. It is shown though solving all three parameter results in the highest r^2, fixing μ at reasonable values, such as 2, does not degrade the overall result much.

Event Number	Starting Date (Year: 2016)	Approximate Event Time Frame (UTC)	Total Rainfall (mm)	Average Precipitation Rate (mm/hr)	Maximum POH	MEHS (inch)	Dominant Storm Type
**1**	April 17	03:55:00-16:30:00	31.3	3.67	0	-	MCS* with trailing stratiform rain region
**2**	April 18	03:16:00-08:30:00	2.4	1.78	0	-	Stratiform rain
**3**	April 19	21:30:00-01:20:00	9.1	3.87	0	-	North end of an MCS
**4**	April 21	03:23:00-04:58:00	3.0	4.26	0	-	Decaying MCS
**5**	April 26	19:00:00-03:30:00	26.5	8.14	100	2.25	Supercells transitioning toward MCS
**6**	April 29	15:28:00-19:31:00	13.7	8.84	60	0.5	Potential supercells/isolated cells
**7**	May 8	11:10:00-18:54:00	2.4	4.83	0	-	Scattered cells
**8**	May 9	02:31:00-03:45:00	1.9	2.53	0	-	Decaying supercells
**9**	May 9	21:45:00-23:50:00	7.0	12.63	100	1.75	Potential supercells/isolated cells

*MCS: Mesoscale Convective System

Supplementary Table 1 | A summary of the nine observed precipitation events. The approximate event time frame is based on the radar and seismic PSD information (Supplementary Movies). Total rainfall is the average accumulated precipitation for places with over 0.5 mm rainfalls and is calculated based on the radar hourly precipitation rate. The average precipitation rate is calculated similarly to the total rainfall based on the radar instantaneous precipitation rate. Each event may not be continuous and could cover different regions at different times, so the approximate event time frame is longer than the rainfall duration if dividing the total rainfall by the average precipitation rate. Maximum POH and MEHS are acquired from the ground-based radar in the same manner as in Fig. 5. The dominant storm type was manually determined from archived weather radar data that was viewed at the following website: https://www2.mmm.ucar.edu/imagearchive/.

Symbols for the Physical Meaning of Seismic Precipitation Signals		Symbols for Data Processing	
PSD	Power spectral density (Eq. 2)	R	Relative Earth structure response (Main text)
F	Impact force (Eq. 1)	CC_0	Cross-correlation value at zero time (Eq. 13)
G	Displacement Green's function (Eq. 1)	CC_{max}	Maximum cross-correlation value (Eq. 13)
PR	Precipitation rate (Eq. 2)	cc	Correlation coefficient (Eq. 14)
E	Raindrop kinetic energy (Eq. 2)	psd	The time series of PSD at 100-200 Hz (Eq. 13)
S	Combined Earth Structure Response (Eq. 2)	snr	Precipitation window signal-to-noise ratio (Eq. 16)
N	Number of raindrops per area per time (Eq. 2)	T	Precipitation window length (Eq. 16)
u	Seismic displacement (Eq. 1)	w	Weighting function (Eq. 15)
f	Seismic frequency (Eq. 1)	$\mathfrak{w}$	Weight for station pair PSD difference measurements (Eq. 16)
r	Distance between raindrop impact and seismic station (Eq. 1)	W	Summed weight for each station pair (Eq. 17)
m	Raindrop mass (Eq. 1)	$r_{i,k}$	Overall measured PSD difference between stations i and k (Eq. 17)
v	Raindrop fall speed (Eq. 1)	$std_{i,k}$	Standard deviation for measured $\log(PSD_i/PSD_k)$ (Eq. 17)
t	Time (Eq. 1)	$\mathcal{R}$	Log-scale R (Eq. 17)
t_j	Time for impact j (Eq. 1)	J	Cost function (Eq. 17)
ρ_w	Raindrop density (Eq. 2)	$\mathbf{R}$	The vector of (Eq. 17)
ρ_a	Air density (Eq. 5)	$\mathbf{g}$	Gradient of the cost function (Eq. 18)
$N(D)$	Number of raindrops per area per time per diameter (Eq. 4)	$\mathbf{H}$	Hessian of the cost function (Eq. 19)
N_0	Characteristic number of raindrops per area per time (Eq. 4)	$\mathcal{W}$	Weight for spatial averaging (Eq. 21)
D	Raindrop diameter (Eq. 4)	σ	Standard deviation of $\mathcal{R}$ (Eq. 21)
D_0	Characteristic raindrop diameter (Eq. 4)	$noise$	Noise level for precipitation event (Eq. 21)
μ	Shape parameter for $N(D)$ (Eq. 4)	d	Distance from station to the location for spatial averaging (Eq. 21)
g	Gravitational acceleration (Eq. 5)	$psdl$	Log-scale psd
i, k	Indices for stations	p, q	Free parameters for linear regression (Eq. 24)
j	Index for impact	m, n	Indices for $\mathbf{R}$, $\mathbf{g}$, and $\mathbf{H}$

Supplementary Table 2 | The summary of symbols used in this study. Symbols appeared in both the Main text and Methods are included. Where these symbols first appear in equations are also indicated.

Supplementary Movie 1 | Precipitation spatial evolution for the event on 17 April 2016. Top row from left to right shows the log-scale seismic PSD (as in Fig. 4a); seismic converted precipitation rate with the relationship in Fig. 3e (as in Fig. S3a); seismic converted one-hour precipitation accumulation (as in Fig. S3b); the current time (red line) and the satellite precipitation rate for the study area (blue line). The bottom row from left to right shows the log-scale radar instantaneous precipitation rate (as in Fig. 4b); radar instantaneous precipitation rate (as in Fig. S3d); radar one-hour precipitation accumulation without gauge-radar bias (as in Fig. S3e); the difference between seismic PSD and radar instantaneous precipitation rate (as in Fig. 4c). For the first three columns, white lines in the top row show contour derived in the bottom row at the value indicated in the panel, and vice versa. Contour levels for seismic PSD are converted to precipitation rate using the formula in Fig. 3e. Radar images are updated more slowly due to lower time resolution. The software to create all the supplementary movies (in map views) is MATLAB, version R2021b (https://www.mathworks.com/products/matlab.html).

Supplementary Movie 2 | Precipitation spatial evolution for the event on 18 April 2016. Similar to Movie S1, but for a different event.

Supplementary Movie 3 | Precipitation spatial evolution for the event on 19 April 2016. Similar to Movie S1, but for a different event.

Supplementary Movie 4 | Precipitation spatial evolution for the event on 21 April 2016. Similar to Movie S1, but for a different event.

Supplementary Movie 5 | Precipitation spatial evolution for the event on 26 April 2016. Similar to Movie S1, but for a different event.

Supplementary Movie 6 | Precipitation spatial evolution for the event on 29 April 2016. Similar to Movie S1, but for a different event.

Supplementary Movie 7 | Precipitation spatial evolution for the event on 08 May 2016. Similar to Movie S1, but for a different event.

Supplementary Movie 8 | Precipitation spatial evolution for the first event on 09 May 2016. Similar to Movie S1, but for a different event.

Supplementary Movie 9 | Precipitation spatial evolution for the second event on 09 May 2016. Similar to Movie S1, but for a different event.
